# A cross-cultural investigation of people’s intuitive beliefs about the origins of cognition

**DOI:** 10.3389/fpsyg.2022.974434

**Published:** 2022-11-08

**Authors:** Xianwei Meng, Jinjing Jenny Wang, Yuichiro Yoshikawa, Hiroshi Ishiguro, Shoji Itakura

**Affiliations:** ^1^Graduate School of Human Sciences, Osaka University, Osaka, Japan; ^2^Department of Psychology, Rutgers University - New Brunswick, New Jersey, NJ, United States; ^3^Graduate School of Engineering Science, Osaka University, Osaka, Japan; ^4^Center for Baby Science, Doshisha University, Kyoto, Japan

**Keywords:** cross-cultural, cognition, intuitive empiricist, folk belief, epistemology

## Abstract

Nature vs. nurture is an enduring theme of studies of the mind. Past studies on American children and adults have revealed a preference for thinking that even fundamental cognitive abilities documented in human infants and non-human species are late-emerging and reliant on learning and nurture. However, little is known about the generalizability of this “intuitive empiricist” belief and what factors may help explain it. Adult participants (*N* = 600) reported their beliefs about the emergence of several fundamental cognitive abilities demonstrated by preverbal infants. Studies 1A-1C showed that adults from both Japan and the US similarly estimated an older age of onset for cognitive abilities in human children as compared to the findings of cognitive science and consistently attributed acquisition of these abilities to learning rather than innateness in humans, and they made these learning attributions more so for humans than for non-human species. Study 2 showed that participants’ beliefs about biological evolution versus creationism were related to their age onset estimates for fundamental cognitive abilities, and their beliefs about the malleability of intelligence were related to participants’ explanations of the origin of fundamental cognitive abilities. These findings suggest generalizable preferences for nurture over nature across both Eastern and Western cultures (Japan and the United States), which may be related to people’s beliefs about human origins and the power of learning.

## Introduction

For millennia, the origins of human thinking have fascinated the world’s greatest thinkers. To what extent is knowledge derived from experience and observations, and to what extent is knowledge endowed by nature and biological inheritance? Eastern and Western traditions alike have produced philosophical work that supported both nativist and empiricist stances since the Buddha, Confucius, Plato, and Aristotle (Locke, 1847; Confucius, 1980; Cooper and Hutchinson, 1997).

In recent decades, experimental studies on the initial stages of the human mind have begun to provide empirical evidence relevant to this debate. When presented with carefully controlled events, human infants show sophisticated neural and behavioral responses that are difficult to explain entirely by parameter-free learning mechanisms [e.g., Skinnerian reinforcement learning (Skinner, 1948)]. For instance, infants as young as a few hours old show preferential looking toward visual–spatial arrays of objects that are matched with auditory sequences based on numbers, suggesting an abstract representation of numerosity (Izard et al., 2009). A similar ability to recognize and abstract numerosities has been shown in various non-human species, including fish (Piffer et al., 2012) and insects (Cronin, 2014). Theories propose that from very early in development, humans are equipped with systems of “core knowledge” (e.g., presentation of abstract numerical quantities) and that the emergence of mature human cognition builds on innate and domain-specific learning mechanisms (Fodor, 1983; Spelke and Kinzler, 2007; Carey, 2009).

While understanding the exact mechanism by which nature and nurture underlie human knowledge is a continued mission of cognitive science, it is also a question that people can easily form intuitive beliefs about (Gopnik and Meltzoff, 1997; Pinker, 2004). These intuitive beliefs may be important for natural pedagogy and influence how scientists approach research questions regarding the origins of human cognition (Rosenthal, 1969; Wang and Feigenson, 2019; Berent, 2021).

Recent investigations of intuitive beliefs about knowledge origins in a Western culture (the United States) revealed a systematic preference for nurture over nature. In one series of studies, adults systematically rated cognitive traits (e.g., having a concept of “person”) as less likely to be innate compared to physical and emotional traits, and were hesitant to accept cognitive traits as innate even when presented with evidence for it (Berent et al., 2019). In another series of studies, US adults and children aged 5–8 years were asked about “core knowledge” abilities, such as the ability to approximately tell different quantities apart. Children, lay adults, and academic scholars thought that these “core” cognitive abilities emerge around preschool years as opposed to infancy. Moreover, emergence of these cognitive abilities was primarily attributed to learning and instruction, rather than being innate or from natural maturation (Wang and Feigenson, 2019).

These findings suggest that people as young as 5 years spontaneously form systematic beliefs about the origins of human knowledge; that is, people may be “intuitive empiricists.” How does this intuitive empiricist belief originate? Nativist proposals have suggested that intuitive empiricist beliefs may result from humans’ natural mentalizing ability (Carruthers, 2020), or a conflict between innate dualist and essentialist beliefs (Berent et al., 2019; Berent, 2021). Both proposals predict the universality of intuitive empiricism. Alternatively, these intuitive empiricist beliefs may stem from other aspects of culture, such as religious beliefs about human nature (DeLeeuw et al., 2007) or societal beliefs about the malleability of intelligence (Claro et al., 2016). Although data from previous studies with Hindu adults and US children were in line with nativist proposals for explaining intuitive empiricist beliefs, more evidence is needed for its universality. Additionally, which factors or aspects of culture or folk beliefs account for intuitive empiricist beliefs remain unknown.

This study aimed to explore the mechanism of being “intuitive empiricists” regarding the origins of human cognition. Studies 1A–1C investigated to what extent culture influences intuitive empiricist beliefs. Regarding the approach, Study 1A directly replicated Wang and Feigenson (2019; Experiment 1) main findings with adults in two cultures: an Eastern (Japan) and a Western culture (the United States), and then compared people’s age onset estimates and origin attribution of core cognitive abilities across the two cultures. Study 2 tested the influence of several candidate individual-level factors – the view about creationism versus biological evolution, mindsets concerning how easily human knowledge and cognitive abilities could be changed by learning, and experience of being a parent or guardian – on people’s estimation and explanation of human knowledge origins.

## Study 1A

Although the existence of intuitive empiricism may not depend on specific cultural experiences [one study showed that both adults in the United States and India prefer nurture to nature in human knowledge origins (Wang and Feigenson, 2019)], most previous investigations have been conducted with Western adults, and the extent to which someone believes about the origins of human knowledge may be related to cultural experiences (Berent et al., 2019; Wang and Feigenson, 2019).

One classical cross-cultural factor that motivated the current investigation is that many studies have argued that Western cultures tend to conceptualize knowledge acquisition differently than Asian cultures (Chen and Stevenson, 1995; Chao, 1996). Stankov (2010) suggested that individuals from Confucian cultures, such as China and Japan, are more likely to attribute success to effort rather than ability compared to individuals from Western cultures. Moreover, when asked to rank the relative importance of different factors for children’s academic performance, American parents were significantly more likely to attribute success to innate ability than effort, compared to Japanese and Chinese parents (Stevenson et al., 1990).

Therefore, compared to individuals from Eastern cultures, individuals from Western cultures may be more likely to endorse that human knowledge relies on innate properties than learning. This would imply the hypothesis that individuals from Eastern cultures systematically provide later age onsets for core knowledge and are more likely to endorse learning-based explanations than individuals from Western cultures.

Study 1A aimed to replicate the main findings of Wang and Feigenson (2019; Experiment 1) with adults in Japan and the United States, and aimed to compare performance across the two cultures to test potential cultural differences in intuitive beliefs about knowledge origins.

### Materials and methods

#### Participants

Data were collected from 100 Japanese adults (50 female; M_age_ = 41.8 years, SD = 8.92), and 100 American adults (59 female; M_age_ = 34.9 years, SD = 11.50). The sample size of 100 was determined according to Wang and Feigenson (2019) recommendations. Participants in Japan were recruited *via* NEO MARKETING (a major Japanese Internet survey company) and US participants were recruited *via* Amazon Mechanical Turk. Data collection stopped when the amount of available data met the planned sample size. Prior to accessing the survey, participants read an informed consent statement stating that participation was voluntary and that they could stop at any time. By clicking on an “agree” button, participants were informed that they were providing consent to complete the survey. The procedure was approved by the Doshisha University Research Ethics Committee (No. 20022).

#### Design, stimuli, and coding

Stimuli were adapted from Wang and Feigenson (2019; Experiment 1). For the investigation in Japan, we translated the questionnaire into Japanese by an English-Japanese bilingual speaker. Then, both the original and translated versions were translated into a third language (i.e., Chinese) by an English-Chinese bilingual speaker and a Japanese-Chinese bilingual speaker to assess reliability. Minor differences in phrasing and vocabulary were reconciled by the three translators.

Participants were first introduced to a character named Alex (Hikaru in Japanese), with photographs illustrating different things Alex/Hikaru can do. Participants were presented with different abilities described with intuitive scenarios, such as “When Alex/Hikaru sees someone hold an object and then drop it, Alex/Hikaru thinks the object will fall.” They were then asked to estimate when they thought each ability first appeared by choosing from images depicting Alex/Hikaru as a newborn, older infant, toddler, preschool child, school-age child, adolescent, or adult. Alex was presented using the same images as in Wang and Feigenson (2019), and Hikaru was presented using images of Japanese age- and sex-matched individuals. After estimating the age of onset, participants were asked to type a free response to the question of why Alex/Hikaru have the ability. Participants were asked about seven core knowledge abilities (color perception: previous studies have observed this ability at 4 months (Bornstein et al., 1976); depth perception: at 2 days (Slater et al., 1990); face recognition: at 168 h (Farroni et al., 2005); physical reasoning: at 3 months (Baillargeon, 1995); object permanence: at 3.5 months (Baillargeon, 1987); approximate numerical discrimination: at 49 h (Izard et al., 2009); social evaluation: at 6 months (Hamlin et al., 2007) and three anchoring abilities [reading: at 6 years in Japan and 7 years in the United States (Hasbrouck and Tindal, 2006; Benesse Educational Research and Development Institute, 2013; The Ministry of Education, Culture, Sports, Science and Technology, M, 2017); seeing: in newborns (Brown and Yamamoto, 1986); and hearing: before birth (Northern and Downs, 2002); please see Supplementary Table S1 for the exact wording of these prompts]).

**Figure 1:**
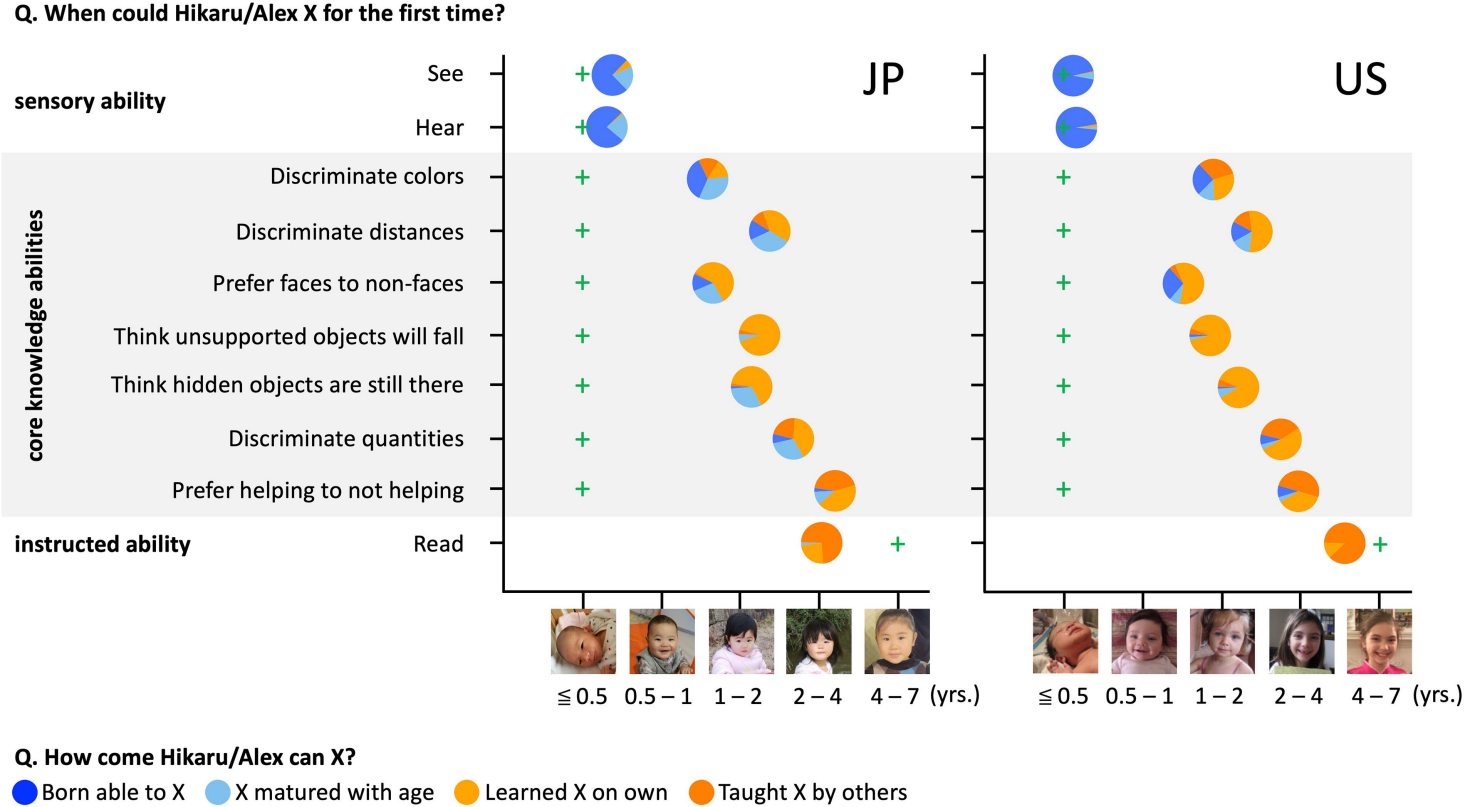
Methods and results for Study1A. Circles’ left–right positions represent participants’ age estimates. Circles’ colors represent the distribution of free responses. Green crosses indicate the earliest age category at which abilities have been documented in published research.

Data coding and analysis were the same as in Wang and Feigenson (2019). We quantified participants’ timeline choices by first estimating Alex/Hikaru age in each timeline image using the midpoint between the labeled lower and upper age bounds (Figure 1). We fit these ages using their ordinal positions, resulting in the function y = 0.13e^0.78x^, R^2^ > 0.99. Using this equation, we translated participants’ timeline responses into an average age of onset estimate for each ability, allowing us to compare participants’ estimates to the average onset age suggested by published findings.

Participants’ free responses to the question “why Alex/Hikaru can X” were coded according to Wang and Feigenson (2019). Two independent coders judged whether each explanation belonged to one of the following four categories: Innateness: this category included explanations that an ability “was innate” or due to biological structures (e.g., “she can see because she has eyes,” “it’s in her genes”), or that a person “was just born able to X.” Maturation: this category included explanations that an ability “emerged on its own” or “happens as a person gets older.” Learning without explicit instruction: this category included explanations that a person “became able to X through their own observations,” or “learned X on their own.” Explicit teaching: this category included explanations that an ability “was taught by others” or “was learned in school.”

The percentage of explanation responses provided by participants that did not clearly fall into any category (e.g., “kids have this ability at this age,” “she is smart”) and were excluded from analysis was 35.4% in the Japanese investigation and 15.7% in the American investigation. Inter-coder reliability was calculated after another coder re-coded 60% of the data: for the Japanese sample, Cohen’s Kappa = 0.916, *p* < 0.001; for the US sample, Cohen’s Kappa = 0.744, *p* < 0.001.

### Results and discussion

#### Response of Japanese adults

To investigate how participants generally estimate the age of onset for core knowledge abilities and reduce the number of comparisons we conduct to reduce Type I error, we averaged the estimations for the seven abilities and compared them to the averaged onset of age suggested by published findings.^1^ Participants estimated that core knowledge abilities emerged, on average, between 1.36 (“Discriminate colors”; 95% CI [1.09, 1.62]) and 4.25 (“Prefer helping to not helping”; 95% CI [3.61, 4.88]) years of age (Figure 1; M_all the core abilities_ = 2.48, SD = 1.71), significantly older than the average age of onset suggested by published findings (M_empirical_ = 0.198; t_(99)_ = 13.35, *p* < 0.001). Participants overwhelmingly explained the core abilities using learning-based explanations (explanations belonged to categories of *Learning without instruction* and *Explicit teaching*) at levels exceeding chance (M = 77%; 95% CI [72, 82%]; t_(96)_ = 10.76, *p* < 0.001, compared to 50% chance when combining the *Innateness* and *Maturation* categories). For seeing and hearing, adults correctly believed that infants do both (M = 0.42 years; 95% CI [0.37, 0.47]; Brown and Yamamoto, 1986; Northern and Downs, 2002) and almost never offered learning-based explanations (M = 4%; 95% CI [−1, 8%]). For reading, adults believed that this ability appears during the preschool period (M = 3.43 years; 95% CI [3.09, 3.78]), earlier than the age of onset suggested at 6 years in Japan (Benesse Educational Research and Development Institute, 2013; The Ministry of Education, Culture, Sports, Science and Technology, M, 2017), and 98% provided learning-based explanations (95% CI [94, 101%]).

The Japanese adults reported an earlier onset of child reading than the researchers do for reading in Japan. This might be because that also scientific investigations have shown that from 6 years old more than half of children can read books by themselves, with an understanding of the words (*Hiragana*) in the books, but from around 4 years old, children show interest in reading even though they cannot well understand the words in the books (Benesse Educational Research and Development Institute, 2013; The Ministry of Education, Culture, Sports, Science and Technology, M, 2017). The adults may have provided an earlier age of onset for reading than the scientific literature shows if they merely considered when children begin to behaviorally “read” books but not whether children can interpret the written words in books.

#### Response of Us adults

Participants estimated that core knowledge abilities emerged, on average, between 1.33 (“Prefer faces to non-faces”; 95% CI [1.08, 1.59]) and 3.49 (“Prefer helping to not helping”; 95% CI [3.09, 3.90]) years of age (Figure 1; M_all the abilities_ = 2.22, SD = 1.00), significantly older than the average age of onset suggested by published findings (M_empirical_ = 0.198; t_(99)_ = 20.28, *p* < 0.001). Participants overwhelmingly explained the core abilities using learning-based explanations (explanations belonged to categories of *Learning without instruction* and *Explicit teaching*) at levels exceeding chance (M = 77%; 95% CI [73, 82%]; t_(97)_ = 11.89, *p* < 0.001, compared to 50% chance when combining the *Innateness* and *Maturation* categories). For seeing and hearing, adults correctly believed that infants do both (M = 0.40 years; 95% CI [0.25, 0.55]) and almost never offered learning-based explanations (M = 3%; 95% CI [0, 6%]). For reading, adults believed that this ability appeared around early school age (M = 5.08 years; 95% CI [4.71, 5.46]), earlier than the age of onset suggested at 7 years in the United States (Hasbrouck and Tindal, 2006), and 100% gave learning-based explanations.

#### Effect of culture on knowledge origin beliefs

**Figure 2:**
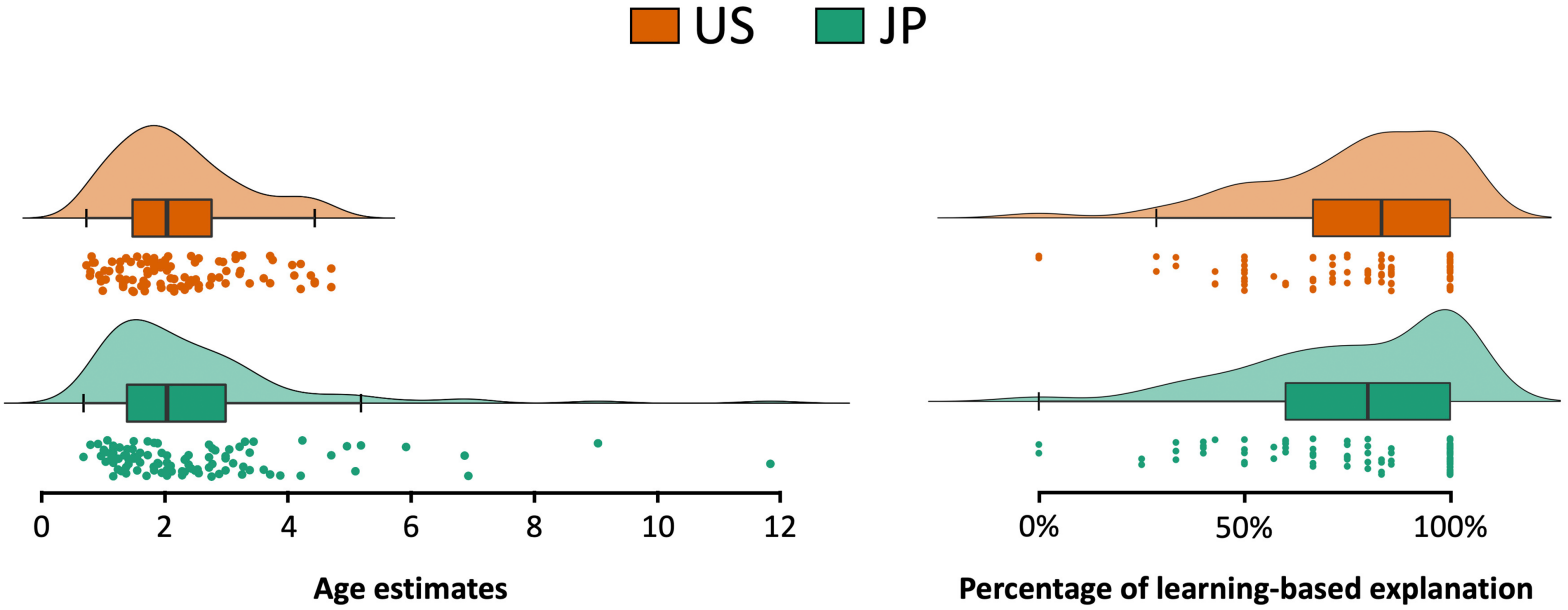
Raw data of participants’ estimated age onset for core ability emergence was presented as raincloud plots (jittered raw data, box plots, and split violin plots). Left and right plots depict data of age estimates of core ability emergence and the percentage participants used learning-based explanation on core ability emergence, respectively.

Participants’ responses were compared to test whether cultural differences could be observed in their beliefs about core ability acquisition. An independent samples t-test demonstrated that Japanese and US adults did not estimate differently the age when core knowledge abilities emerge (t_(159.3)_ = 1.31, *p* = 0.191, *d* = 0.186; 95% CI [−0.093, 0.463], Figure 2). A non-significant response pattern was also observed for sensory abilities, which included seeing and hearing (t_(120.4)_ = 0.23, *p* = 0.818, *d* = 0.033; 95% CI [−0.245, 0.310]), whereas Japanese adults estimated that humans could read at an earlier stage than US adults did (t_(197.0)_ = 6.40, p < 0.001, *d* = 0.905; 95% CI [0.613, 1.195]).

In addition, to test for the absence of a meaningful effect of culture on age estimates of core abilities, we used a two one-sided tests (TOST) procedure to test for equivalence and reject the presence of the smallest effect size of interest (SESOI; Rogers et al., 1993; Lakens et al., 2018). Wang and Feigenson (2019) showed that most participants tend to estimate the age by choosing a picture between the second one (0.5–1 years old) and the fifth one (4–7 years old; Figure 1). Given that the smallest age difference between two often-chosen pictures is 0.75 (i.e., the difference between the mean of the second picture and the third picture), we set the SESOI as a raw mean difference of 0.75 years old. The TOST procedure for Welch’s *t* test for independent samples showed that the effect of culture was statistically equivalent (*p*_upper bound_ = 0.007, *p*_lower bound_ < 0.001).

There was no cultural difference in the percentage that the participants applied learning-based explanations regarding core knowledge abilities (t_(191.9)_ = 0.05, *p* = 0.960, *d* = 0.007; 95% CI [−0.065, 0.069]), sensory abilities (t_(156.13)_ = 0.42, *p* = 0.678, *d* = 0.063; 95% CI [−0.040, 0.061]), or reading ability (An independent samples t-test could not be conducted because the variance in response of the US adults was equal to 0. Note that participants in the two cultures gave almost the same responses: 81/83 Japanese adults and 97/97 US adults applied learning-based explanations). Equivalence testing was used to test the absence of a meaningful effect of culture on participants’ tendency to apply learning-based explanations. Although we did not have theoretical references for determining the SESOI, we set it to a medium effect size of Cohen’s *d* = 0.5 (Cohen, 1992). The TOST procedure for Welch’s *t* test for independent samples showed that the effect of culture was statistically equivalent (*p*_upper bound_ < 0.001, *p*_lower bound_ < 0.001).

Study 1A replicated Wang and Feigenson (2019) in adults in Japan and the US. Both systematically provided later age onsets for core knowledge abilities compared to the empirical findings. Moreover, participants from both cultures were more likely to attribute the origins of their core knowledge abilities to learning and experience. Importantly, there were no cultural differences in these responses, suggesting that intuitive empiricist beliefs are held to a stable degree across the United States and Japan.

Two additional studies, following Wang and Feigenson’s experimental design, evaluated the extent of empiricist beliefs in Japanese adults’ responses. Given that Study1A did not find different responses regarding the emergence of core abilities between Japanese and American adults, in the following studies we did not collect data from American adults for comparison.

## Study 1B

Study 1B tested whether the estimated later age onsets for core knowledge was a byproduct of the way we worded the items. As the use of the verbs “tell” and “think” in Study 1A could have connoted metacognitive awareness or verbal proficiency (e.g., “When could Alex/Hikaru tell near and far for the first time?”), Study 1B used descriptions that decreased the emphasis on metacognitive or verbal capacities (e.g., “When could Hikaru first reach more for close-by things than far-away things?”; please see Supplementary Table S2 for the exact wording of these prompts).

### Materials and methods

#### Participants

Data were collected from 100 Japanese adults (52 women; M_age_ = 42.0 years, SD = 8.76). The data collection procedure was identical to that used in Study 1A.

#### Design, stimuli, and coding

Stimuli were adapted from Wang and Feigenson (2019; Experiment 1c). The translation process of the questionnaire was identical to that of Study 1A. Design and stimuli were as in Study 1A, with modified wordings: Adults saw abilities described in terms of behaviors (e.g., “When could Hikaru first reach more for close-by things than far-away things?”). Coding and analyses were as in Study 1A. The percentage of explanation responses provided by participants that did not clearly fall into any category and were excluded from analysis was 41.7%. The inter-coder reliability of 60% of the free responses was Cohen’s kappa = 0.826, *p* < 0.001.

### Results and discussion

Participants estimated that core knowledge abilities emerged, on average, between 1.21 (“Discriminate colors”; 95% CI [1.03, 1.39]) and 5.32 (“Prefer helping to not helping”; 95% CI [4.31, 6.32]) years of age (Mean_all the core abilities_ = 2.56, SD = 1.60), significantly older than the average age of onset suggested by published findings using looking time methods (M_empirical_ = 0.198; t_(99)_ = 14.73, *p* < 0.001). These age onset estimates are also significantly older if we adjust the comparison points to account for infants’ motor development [e.g., that infants do not reliably reach for hidden objects until 9 months of age (Piaget, 1954); Infants reach for larger quantity by 10 months (Feigenson et al., 2002); ps < 0.001; Supplementary Table S6]. Moreover, there was no significant difference between the age onset estimation for fundamental cognitive abilities (the average of the age estimation of the seven core abilities) in Study 1A and 1B (t_(88)_ = −0.32, *p* = 0.752).

Participants explained the behaviors using learning-based explanations (explanations belonged to categories of *Learning without instruction* and *Explicit teaching*) at chance (M = 48%; 95% CI [40, 55%]; t_(88)_ = −0.68, *p* < 0.498, compared to 50% chance when combining the *Innateness* and *Maturation* categories). For seeing and hearing, adults correctly believed that infants do both (M = 0.37 years; 95% CI [0.34, 0.40]) and almost never offered learning-based explanations (M = 1%; 95% CI [−1, 2%]). For reading, adults believed that this ability appears around early school age (M = 3.27 years; 95% CI [2.84, 3.70]), and 90% gave learning-based explanations (95% CI [83, 96%]).

Study 1B demonstrated that the estimated later age onsets for core knowledge in Study 1A could not be explained as a byproduct of the way the participants worded the items. This is because a similar estimation pattern was found in Study 1B, in which the questions did not include words that would connote metacognitive awareness or verbal proficiency. However, when asked to explain the behaviors in Study 1B, the participants did not show a preference for learning-based reasons. This is different from what was found in Wang and Feigenson, 2019. One possibility is that Japanese participants may have interpreted the ability descriptions differently, such that they tried to explain the ability to perform actions (e.g., if they respond “they grew bigger,” it would have been coded as through biological maturation). Indeed, participants in Study 1B provided responses belong to the category of “X matured with age” more frequently that Study 1A (Supplementary Tables S5–S6). Relatedly, it is possible that this response pattern is a by-product of the different ways in which the questions were phrased in Japanese “Naze?” – which more directly translates to “Why,” compared to in English “How come” – which can be interpreted as “how did it come to be.” As a result, Japanese participants were more likely to provide responses that did not clearly fall into any of our pre-defined categories than the US participants. Nevertheless, this did not seem to influence their estimation of the age when they think the behaviors emerge.

## Study 1C

Study 1C tested whether Japanese adults hold empiricist beliefs about human but not animal abilities. Confirming that intuitive empiricist beliefs are specific to human abilities would confirm that this pattern was not a methodological artifact.

### Materials and methods

#### Participants

Data were collected from 200 Japanese adults (91 women; M_age_ = 42.4 years, SD = 9.62). Half of the participants were assigned to the human condition, and the other half were assigned to the animal condition. The data collection procedure was identical to that in Study 1A.

#### Design, stimuli, and coding

**Figure 3:**
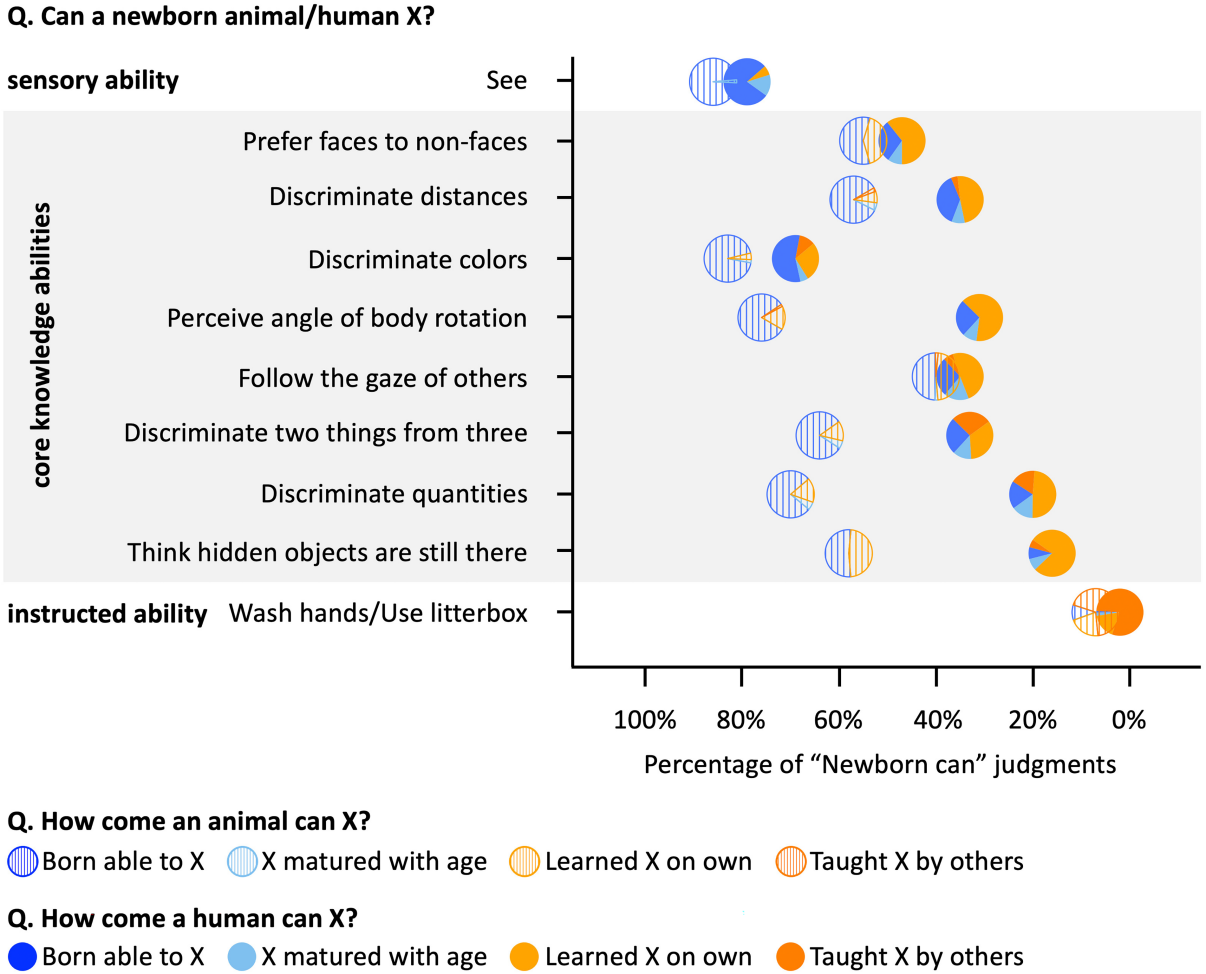
Methods and results for Study 1C. Circles’ left–right positions represent percentage that participants judged the corresponding abilities exist in newborns. Circles’ colors represent the distribution of free responses.

Stimuli were adapted from Wang and Feigenson (2019; Experiment 3). The translation process of the questionnaire was identical to that of Study 1A. Participants were asked to report whether they believed a given ability to be present at birth and describe its origins using free responses (Figure 3). The critical test items were core perceptual and cognitive abilities demonstrated in both humans and animals: humans/chickens discriminating faces of conspecifics from nonfaces (Farroni et al., 2005; Rosa-Salva et al., 2009), humans/spiders discriminating nearby from distant objects (Slater et al., 1990; Nagata et al., 2012), humans/salamanders discriminating colors (Bornstein et al., 1976; Przyrembel et al., 1995), humans/ants discriminating angles of rotation (Landau and Spelke, 1988; Muller and Wehner, 1988), humans/crows following others’ gaze (Johnson et al., 1998; Schloegl et al., 2007), humans/bees discriminating two objects from three (Starkey and Cooper, 1980; Gross et al., 2009), humans/fish discriminating approximate numerosities (Agrillo et al., 2008; Izard et al., 2009), and humans/chickens thinking that a hidden object still exists (Baillargeon, 1987; Vallortigara et al., 1998). The anchor abilities were chosen to elicit agreement that learning was either not required (i.e., seeing in humans/horses) or was required (i.e., washing hands in humans/using a litterbox in cats; please see Supplementary Tables S3, S4 for the exact wording of these prompts).

As in previous studies, the way the abilities were described may have contributed to how participants responded to the questions. To account for potential differences in participants’ perception of animal age vs. human age (e.g., 5 years in a chicken’s life is much older than in a human’s life), participants were asked to indicate whether a newborn human/animal could do something, instead of providing a numeric age estimate or pointing at a picture scale.

Even though we tried to match the abilities as closely as possible between humans and animals, there are inevitably differences in the descriptions and how people interpret these abilities. For example, washing hands is not quite the same as using a litterbox, and the item for “body rotation” for ants requires the ant to move on its own, whereas the same item for humans was to be spun around. Wherever possible, these abilities were made to sound “harder” in animals than humans – which is the opposite to our hypothesis that participants should ascribe *less* learning to animals than humans.

Coding and analysis were performed as described in Study 1A. The percentage of explanation responses provided by participants that did not clearly fall into any category and were excluded from analysis was 44.6%. Inter-coder reliability for all free responses was Cohen’s kappa = 0.887, *p* < 0.001.

### Results and discussion

People’s intuitions about the anchor abilities were similar for humans and animals: adults reported seeing as present at birth in both species (M_human_ = 79%; M_horse_ = 86%) and almost never offered learning-based explanations (human M = 7%; horse M = 0%; Figure 3). They believed that the behavior of washing hands/using a litterbox is not present at birth in either humans or cats (human M = 2%; cat M = 7%), and offered mostly learning-based explanations (human M = 97%; cat M = 90%). Critically, we found that people’s intuitions diverged for the core ability items; they were significantly less likely to endorse core abilities as present in newborn humans (M = 36%; 95% CI [30, 42%]) than newborn animals (M = 63%; 95%CI [58, 68%]; t(198) = 6.88, *p* < 0.001), and offered more learning-based explanations for core abilities in humans (M = 72%; 95% CI [66, 79%]) than in animals (M = 26%; 95% CI[20, 32%]; t(181) = 10.07, *p* < 0.001). Although participants readily explained animals’ abilities by appealing to genes, evolution, and innateness, for the very same abilities in humans, they typically invoked observation and learning. Participants’ responses regarding when and how core abilities emerge in humans and animals were consistent with those of American adults in a previous study (Wang and Feigenson, 2019).

Overall, studies 1A-C replicated the main findings of Wang and Feigenson (2019) with adults in Eastern (Japan) and Western (the United States) cultures, and showed that the bias of overestimating the age of human core abilities and explaining their emergence with learning-based explanations are qualitatively similar in Japan and the United States. However, it is still unclear what factors contribute to intuitive empiricist beliefs. In Study 2, we explored the potential mechanisms by testing the influence of various possible factors.

## Study 2

In Study 2, we tested several individual-level candidate factors that may influence people’s estimation and explanation of the emergence of human core abilities. One candidate might be their view of human origins (DeLeeuw et al., 2007). Two typically contrastive views – creationism and naturalistic evolutionism – can be observed to be prevalent in most regions around the world. Creationism posits that new kinds of species can be created by supernatural acts of divine creation, whereas the naturalistic theory of evolution emphasizes the continuity of species and posits that changes over time are due to random mutation and genetic drift. People who have a stronger evolutionary view of human origins would be more likely to believe that, like other animals (as shown in Study 1C), human cognition has a deep (at the genetic level) developmental origin. Thus, we hypothesized that they would predict that human core abilities emerge at an earlier stage and that they are less likely to provide learning-based explanations regarding the emergence of core abilities.

Another candidate factor would be people’s mindsets of how easily human knowledge and cognitive abilities can be changed by learning. Numerous studies have shown individual differences in people’s mindset beliefs regarding the malleability of intelligence, which plays a moderating role in facilitating academic outcomes (Claro et al., 2016). Studies 1A—1C demonstrated that both Japanese and American adults tend to treat core cognitive abilities as outcomes of learning processes. Therefore, it might be that people who have greater agreement that knowledge and cognitive abilities can be easily changed by learning would predict that core human abilities emerge at an earlier stage. Additionally, they would be more likely to provide learning-based explanations regarding the emergence of core abilities.

The last candidate factor would be the experience of being a parent or guardian. This is because observing real child development processes would help one understand the development of core abilities in young children and consequently reduce overestimation of the age of core knowledge emergence. Therefore, we hypothesized that people with experience of being parents or guardians would predict that core human abilities emerge at an earlier stage. Moreover, they would be less likely to provide learning-based explanations regarding the emergence of core abilities.

### Materials and methods

#### Participants

Data were collected from 100 Japanese adults (43 women; M_age_ = 45.5 years, SD = 11.75). The data collection procedure was identical to that used in Study 1A.

#### Design, stimuli, and coding

The experimental design and stimuli were identical to those of Study 1A, with the following modifications. First, we included three additional “core” ability items that emerge very early in development – recognition of dominance relationships [previous study has observed this ability at 10 months (Thomsen et al., 2011)], exploration of unexpected events [at 11 months (Stahl and Feigenson, 2015)], and rule-based learning [at 8 months (Saffran et al., 1996)], to draw a fuller picture of people’s beliefs about core ability emergence (Supplementary Table S1).

Second, we included questions to test the influence of candidate factors on core ability emergence predictions. The Human Origins Questionnaire (HOQ) was used to assess participants’ views of human origins (DeLeeuw et al., 2007). The participants endorsed one of four statements concerning the origin and development of human beings, including Naturalistic evolution (i.e., “Human beings have developed over millions of years from earlier species or less advanced forms of life; God had no part in this process”), Intelligent design (i.e., “Human beings have developed over millions of years from earlier species or less advanced forms of life; God guided this process”), Old earth creationism (i.e., “God created human beings pretty much in their present form at some point millions of years ago”), and Young earth creationism (i.e., “God created human beings pretty much in their present form at one time within the last 10,000 years or so”). Each option was assigned a numerical dummy code such that increasing values indicated support for creationism (DeLeeuw et al., 2007).

To test people’s mindsets concerning how easily human knowledge and cognitive abilities can be changed by learning, we used a modified version of Dweck (1999) mindset questionnaire (Dweck, 1999), focusing on the extent to which people have fixed views about crystallized intelligence and fluid intelligence (Sun et al., 2020). The participants read the definitions of crystallized and fluid intelligence, and indicated to which degree they think each type of intelligence could be changed by learning (i.e., “Basically, learning can really do much to improve the amount of crystal intelligence.” 1 = strongly disagree, 6 = strongly agree). A “learning-effectiveness-belief” score was calculated by summing the score representing how much people think that crystallized and fluid intelligence can be improved by learning.

Third, to reduce the task load of explaining the target behaviors, we used forced-choice questions instead of free responses. Participants answered the question “why Alex can X” by choosing one of four selections that each belongs to one of the four categories (see Study 1A). Coding and analyses were as in Study 1A.

### Results and discussion

**Figure 4:**
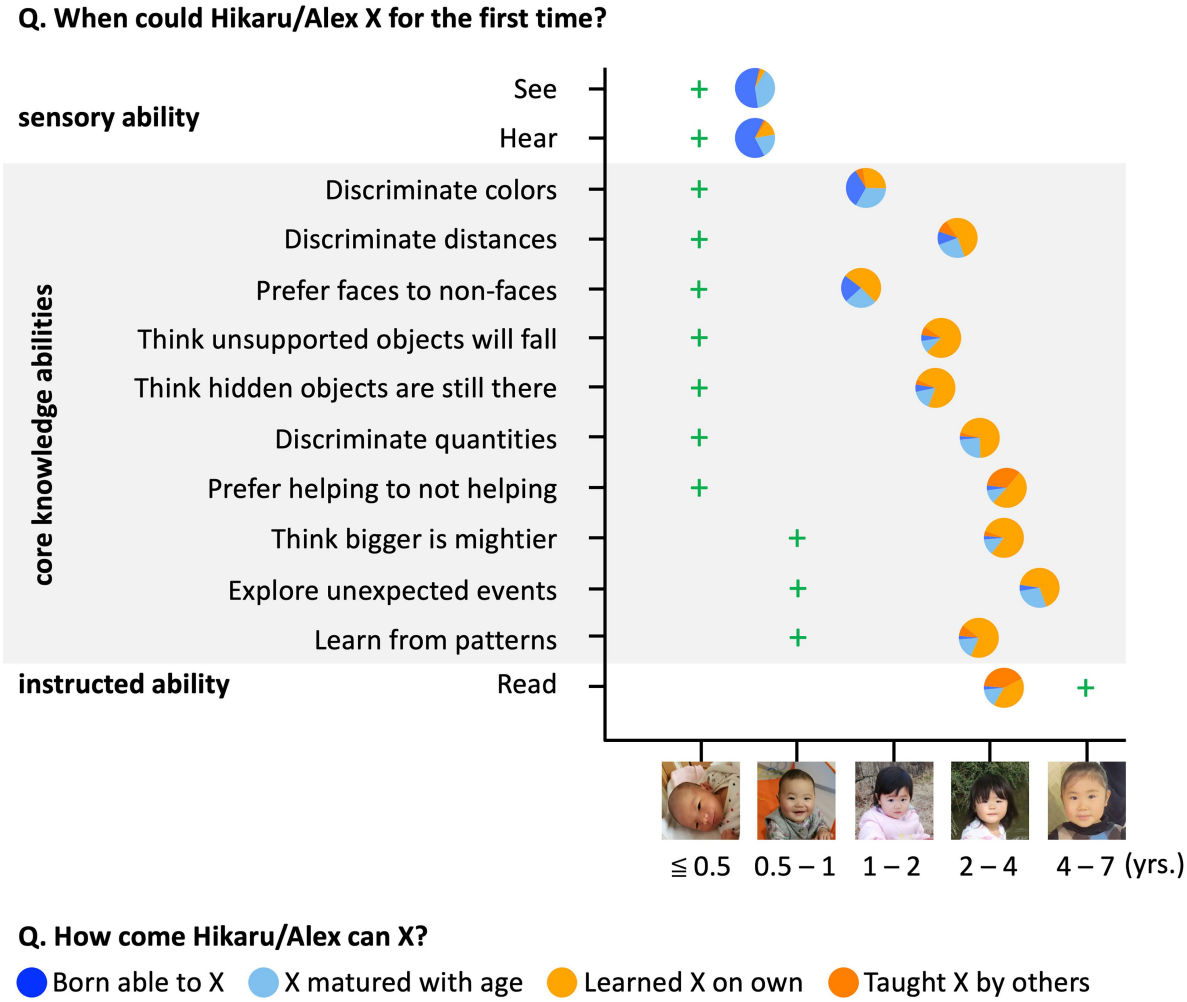
Methods and results for Study 2. Circles’ left–right positions represent participants’ age estimates. Circles’ colors represent the distribution of free responses. Green crosses indicate the earliest age category at which abilities have been documented in published research.

Participants estimated that core knowledge abilities emerged, on average, between 1.55 (“Discriminate color”; 95% CI [1.20, 1.91]) and 5.74 (“Explore unexpected events”; 95% CI [4.95, 6.53]) years of age (M_all the core abilities_ = 3.37, SD = 1.93; Figure 4), significantly older than the average age of onset suggested by published findings (M_empirical_ = 0.380; t_(99)_ = 15.53, *p* < 0.001). Participants tended to explain the behaviors using learning-based explanations (explanations belonged to categories of *Learning without instruction* and *Explicit teaching*) at levels exceeding chance (M = 62%; 95% CI [57, 66%]; t_(99)_ = 5.27, *p* < 0.001, compared to 50% chance when combining the *Innateness* and *Maturation* categories). For seeing and hearing, adults correctly believed that infants do both (M = 0.64 years; 95% CI [0.48, 0.81]) and almost never offered learning-based explanations (M = 10%; 95% CI [5, 15%]). For reading, adults believed that this ability appears around early school age (M = 4.25 years; 95% CI [3.51, 5.00]), and 82% gave learning-based explanations (95% CI [74, 90%]).

A univariate linear regression with participants’ age onset estimates as the outcome variable and gender, age, parental status, creationism, and learning-effective-belief as predictors revealed that creationism was a significant predictor (*β* = 0.54, *t* = 2.78, *p* = 0.007). Gender (*β* = 0.65, *t* = 1.69, *p* = 0.095), age (*β* = −0.03, *t* = −1.52, *p* = 0.132), parental status (*β* = −0.30, *t* = −0.78, *p* = 0.435), and learning-effective-belief (*β* = −0.30, *t* = −1.08, *p* = 0.282) did not predict participants’ age onset estimates.

Univariate linear regression with percentage of learning-based explanations as the outcome variable and gender, age, parental status, creationism, and learning-effective-belief as predictors revealed that learning-effective-belief was a significant predictor (*β* = 0.10, *t* = 2.81, *p* = 0.006). Gender (*β* = 0.03, *t* = 0.69, *p* = 0.493), age (*β* = −0.002, *t* = −0.80, *p* = 0.426), parental status (*β* = −0.04, *t* = −0.83, *p* = 0.406), and creationism (*β* = −0.008, *t* = −0.31, *p* = 0.759) did not predict the percentage of learning-based explanations.

Study 2 replicated the findings of Studies 1A–1C that participants systematically provided later age onsets for core knowledge and tended to attribute the emergence to learning and instruction as opposed to being innate or from natural maturation. Moreover, the study confirmed that one’s views of human origins are related to the age onset estimation of the core ability emergence, and that one’s views about the power of learning are related to one’s explanation of core ability acquisition.

## General discussion

Recent findings in developmental science have shown that some fundamental cognitive abilities (e.g., recognition of numeric presentation) may have deep developmental origins, suggesting early learning-independent structures of human cognition. However, people seem to have intuitive beliefs that are more likely to endorse nurture than nature when explaining the origins of even the most fundamental cognitive abilities. Although these “intuitive empiricist” beliefs may have significant impacts on pedagogical practices and implications for the scientific inquiry of the mind (Rosenthal, 1969; Wang and Feigenson, 2019; Berent, 2021), less has been known about the mechanism about why people are “intuitive empiricists.” By replicating and extending the original findings of Wang and Feigenson (2019), the current study suggests that the degree to which people are “intuitive empiricists” does not differ between Japanese and American adults. Specifically, adults from both cultures, at similar levels, systematically provided later age onsets for core knowledge and preferred learning-based explanations about cognitive ability acquisitions. Moreover, the current study found that biased intuitive beliefs about human knowledge origins might be related to one’s views of human origins and of the power of learning.

This study first successfully replicated the main findings of Wang and Feigenson (2019) with participants from Western and Eastern cultures. We did not find cultural differences – either quantitively or qualitatively – on people’s “intuitive empiricist belief” about human knowledge origins. Given that past studies suggest that, compared to people from Western cultures, people from Eastern cultures are more likely to emphasize effort (instead of innate ability) in intellectual development, it was easy to predict that individuals from Eastern cultures are more likely to endorse that human knowledge relies on learning and experience rather than innate properties. However, our findings did not support this hypothesis. Although future studies should look deeper at cultural differences that may affect people’s beliefs about the origins of human knowledge, our results suggest that such beliefs do not seem to vary across cultures, at least regarding two typical samples from Eastern and Western cultures.

When examining factors that predict individual differences in people’s intuitive beliefs about knowledge origins, the results revealed potential connections between “intuitive empiricist” beliefs and people’s beliefs about human nature and the power of learning. First, the results suggest that the degree of endorsement of creationism is positively related to a later age of onset of knowledge emergence, but the results did not show its influence on the likelihood that people use learning-based explanations about knowledge emergence. This suggests that having beliefs that endorse the continuity of species (and that human cognition may have a deep developmental origin as in other animals) partly contributes to scientific understanding of when human knowledge emerges in ontogeny (however, note that people who believe in evolution still may estimate ages that are significantly later than the science shows). Second, the results suggest that people who more strongly endorse the power of learning are more likely to believe that knowledge is acquired through learning. However, we did not find a relationship between belief in the power of learning and when early knowledge emerges. This pattern of findings suggests that separate psychological processes may underlie people’s estimates of the time at which abilities emerge and their explanations for why the abilities emerge. Specifically, the belief that fundamental human cognitive abilities emerge late in development is correlated with a lack of understanding of evolution, whereas the belief that learning and instruction are required for acquiring fundamental cognitive abilities is correlated with beliefs about how malleable the mind is.

More surprisingly, the experience of being a parent or guardian did not relate to either belief about when knowledge emerges or belief about how knowledge is acquired. This might be because, in daily life, there is not much chance to observe infants’ responses in specific contexts, which would make parents or guardians believe that infants are capable of understanding the contexts. For instance, even though infants may often see a ball falling to the ground, they rarely see balls remaining suspended without physical support – an event that would violate their expectations so that the infants might show expectation-violated responses (e.g., paying attention to the events and exploring the reason why the ball can remain suspended; Baillargeon and Hanko-Summers, 1990; Stahl and Feigenson, 2015; Meng et al., 2021). Although infants demonstrate sensitivity and competence in various cognitive domains when tested in carefully designed in-lab experiments, the types of measures used by scientists are opaque to the naked eye, such as differences in infants’ looking time or infants’ brain activity. People’s intuitive beliefs about knowledge origin may reflect the emergence of children’s easily demonstratable skills in these domains in naturalistic and somewhat uncontrolled environments (e.g., Piaget and Inhelder, 2008). Future studies should attempt to explain the possible distinctions between people’s subjective beliefs about human development and objective (scientific) facts of human development.

Several limitations of this study, which lead to other key questions for future research, should be noted. Although overall participants in the United States and Japan showed similar patterns of results, their responses may differ in more subtle ways. First, the percentage of explanation responses provided by participants that did not clearly fall into any pre-defined category, and which were excluded from analysis, was 35.4% in the Japanese sample vs. 15.7% in the American sample (Japanese samples also show relatively high exclusion rates for Study 1B and Study 1C). This difference was most likely a result of the slightly different interpretation of the English question “How come …?” compared to the Japanese question “Naze?” – which more directly translates to “Why?” Compared to “How come” – which can be interpreted as “how did it come to be” - “why” can be answered from a wider range of perspectives, such as what mechanism or function an ability has. When we translated Wang & Feigenson’s English questionnaire into a Japanese questionnaire, we could not find a Japanese question that conveys the same nuance as the English question of “How come...?.” Therefore, we used “Naze?.” As a result, Japanese participants were more likely to provide responses that did not fall into any of our pre-defined categories than the US participants. Note that, even though the “Naze” question may have led Japanese participants to be less likely to mention ontogeny of the abilities compared to the US sample, our finding showed that within the responses that were relevant to ontogeny, there was no significant difference between Japanese sample and the US sample, both of which consistently attributed acquisition of the abilities to learning rather than innateness in humans. We also found no difference in their estimates for when core cognitive abilities emerge in development. Nevertheless, further studies should take a deeper look at how the semantics and pragmatics of questions may influence people’s intuitive beliefs about the origins of cognition.

Second, question phrasing may influence participants from the US and Japan differently. In Wang and Feigenson (2019), changes in the way the different core cognitive abilities were described did not significantly influence US participants’ responses to either the age of onset for the abilities or where the abilities came from. In contrast, in this study, we found that Japanese adults in Study 1A offered more learning explanations than chance, but not in Study 1B when the wording of the abilities changed to focus more on the motor actions rather than the mental processes underlying the cognitive abilities. This suggests that Japanese adults’ intuitive explanations for where core cognitive abilities come from are influenced by how the abilities were exactly phrased. Specifically, participants provided more responses that belonged to the “maturation” category, which may also reflect underlying differences in semantic and pragmatic interpretations of the prompts between Japanese and English. However, participants’ estimates for when core cognitive abilities emerge did not differ between Study 1B and Study 1A. These findings suggest that even though the wording may influence participants’ spontaneous explanation of where cognitive abilities come from, it does not determine participants’ perception of the emergence of the abilities during development. Future investigations should explore whether and how age estimation and reasoning for the origin of cognition share the same or different attribution mechanisms.

Another limitation of the present research is that some of the questions presented to the adult participants may have contributed to differences in participants’ estimates for the age of onset versus what has been shown in science (see Supplementary Tables S1–S4 for the wording used for adult participants). For example, in the “helping” item of Experiment 1A, participants were asked about watching someone helping a turtle who was upside down and struggling to get back on its feet. This is different from experiments that revealed infants’ responses to helping vs. hurting, where infants typically watched puppets helping or hurting each other in terms of achieving their goals (Hamlin et al., 2007). This discrepancy in scenarios may have contributed to the difference between participants’ estimates of when the ability emerges compared to what has been revealed by science. On the other hand, other descriptions were closely matched to research paradigms, such as preferring faces to non-faces (Farroni et al., 2005). Therefore, the discrepancy in scenarios alone cannot explain all the differences we have observed across all the experiments. This issue could be further investigated by using wording that more closely matched the scientific experiments.

Finally, this study aimed to provide more insights into the mechanism that causes people’s tendency to overestimate when core cognitive abilities emerge and to favor empiricist explanations. Study 2 provided some initial evidence that people’s beliefs about human nature and the power of learning may underlie this “intuitive empiricist” belief. However, it is possible that people’s beliefs about human nature and the power of learning may be reflecting differences in some other factor. For example, people from different socio-economic or educational backgrounds may hold different beliefs about evolution and creation (Banerjee and Bloom, 2013; Norenzayan and Gervais, 2013), and these broader social-structural factors may be the common cause for all three types of beliefs about human nature, power of learning, and the origins of cognition. It is also possible that other social factors, such as political stance or personality traits, may contribute to people’s intuitive beliefs about the mind. Future research that experimentally manipulates these different factors or longitudinally tracks people’s beliefs along these different dimensions will provide further insights into the causal factors that shape people’s intuitive theories of cognition.

Nature vs. nurture is one of the enduring themes of studies of human knowledge. While developmental science has shown that some aspects of human knowledge have deep developmental origins, our findings suggest a universal preference for nurture over nature across cultures, which may be influenced by beliefs about the origins of the human species and the malleability of the mind. The current findings provide a new wave of research aimed at understanding the nature of human cognition through the lens of intuitive folk beliefs about the mind.

## Data availability statement

The raw data supporting the conclusions of this article will be made available by the authors, without undue reservation.

## Ethics statement

The studies involving human participants were reviewed and approved by Prior to accessing the survey, participants read an informed consent statement stating that participation was voluntary and that they could stop at any time. By clicking on an “agree” button, participants were informed that they were providing consent to complete the survey. The procedure was approved by the Doshisha University Research Ethics Committee (No. 20022). The patients/participants provided their written informed consent to participate in this study. Written informed consent was obtained from the individual(s), and minor(s)’ legal guardian/next of kin, for the publication of any potentially identifiable images or data included in this article.

## Author contributions

XM and JW developed the study concept, performed the data collection and the data analysis, and drafted the manuscript. All authors contributed to the article and approved the submitted version.

## Funding

This work was supported by the research grant of Japan Society for Promotion of Scientific Research (20 K20156), MEXT “Innovation Platform for Society 5.0” Program Grant Number JPMXP0518071489, and MEXT Promotion of Distinctive Joint Research Center Program Grant Number JPMXP0619217850.

## Acknowledments

We thank the reviewers for their careful reading of our manuscript and their many insightful comments and suggestions. We thank Chiaki Kimura, Kimiko Uenoyama, Clara Angioletti, Amy Xu, and Honami Meng for their help with the materials and coding.

## Conflict of interest

The authors declare that the research was conducted in the absence of any commercial or financial relationships that could be construed as a potential conflict of interest.

## Publisher’s note

All claims expressed in this article are solely those of the authors and do not necessarily represent those of their affiliated organizations, or those of the publisher, the editors and the reviewers. Any product that may be evaluated in this article, or claim that may be made by its manufacturer, is not guaranteed or endorsed by the publisher.

## Supplementary material

The Supplementary material for this article can be found online at: https://www.frontiersin.org/articles/10.3389/fpsyg.2022.974434/full#supplementary-material

## Footnotes

^1^To draw a fuller picture of participants’ estimations, we also separately tested whether participants estimated an older onset of age for each ability than that suggested by published findings (See Supplementary Tables S5–S8).

## References

[ref1] Agrillo C. Dadda M. Serena G. Bisazza A. (2008). Do fish count? Spontaneous discrimination of quantity in female mosquitofish. Anim. Cogn. 11, 495–503. doi: 10.1007/s10071-008-0140-9, PMID: 18247068

[ref2] Baillargeon R. (1987). Object permanence in 3½-and 4½-month-old infants. Dev. Psychol. 23, 655–664. doi: 10.1037/0012-1649.23.5.655

[ref3] Baillargeon R. (1995). Physical reasoning in infancy. The Cognitive Neurosciences, ed. M. S. Gazzaniga, MIT Press. 181–204.

[ref4] Baillargeon R. Hanko-Summers S. (1990). Is the top object adequately supported by the bottom object? Young infants’ understanding of support relations. Cogn. Dev. 5, 29–53. doi: 10.1016/0885-2014(90)90011-H

[ref5] Banerjee K. Bloom P. (2013). Would Tarzan believe in god? Conditions for the emergence of religious belief. Trends in cognitive sciences 17, 7–8. doi: 10.1016/j.tics.2012.11.005 23238119

[ref6] Benesse Educational Research and Development Institute (2013). 第1回 幼児期から小学1年生の家庭教育調査報告書. Available at: https://berd.benesse.jp/jisedai/research/detail1.php?id=3200

[ref7] Berent I. (2021). Can we get human nature right? Proc. Natl. Acad. Sci. U. S. A. 118, 28–31. doi: 10.1073/pnas.2108274118, PMID: 34556578 PMC8488640

[ref8] Berent I. Platt M. Sandoboe G. M. (2019). People’s intuitions about innateness. Open. Mind 3, 101–114. doi: 10.1162/opmi_a_00029 PMC8412331 34485790

[ref9] Bornstein M. H. Kessen W. Weiskopf S. (1976). Color vision and hue categorization in young human infants. J. Exp. Psychol. Hum. Percept. Perform. 2, 115–129. doi: 10.1037/0096-1523.2.1.115, PMID: 1262792

[ref10] Brown A. M. Yamamoto M. (1986). Visual acuity in newborn and preterm infants measured with grating acuity cards. Am J. Ophthalmol. 102, 245–253. doi: 10.1016/0002-9394(86)90153-4, PMID: 3740187

[ref11] Carey S. (2009). Where our number concepts come from. J. Philos. 106, 220–254. doi: 10.5840/jphil2009106418, PMID: 23136450 PMC3489488

[ref12] Carruthers P. (2020). How mindreading might mislead cognitive science. J. Conscious. Stud. 27, 195–219.

[ref13] Chao R. K. (1996). Chinese and European American mothers’ beliefs about the role of parenting in children’s school success. J. Cross-Cult. Psychol. 27, 403–423. doi: 10.1177/0022022196274002

[ref14] Chen C. Stevenson H. W. (1995). Motivation and mathematics achievement: a comparative study of Asian-American, Caucasian-American, and east Asian high school students. Child Dev. 66, 1215–1234. doi: 10.1111/j.1467-8624.1995.tb00932.x 7671657

[ref15] Claro S. Paunesku D. Dweck C. S. (2016). Growth mindset tempers the effects of poverty on academic achievement. Proc. Natl. Acad. Sci. U. S. A. 113, 8664–8668. doi: 10.1073/pnas.1608207113, PMID: 27432947 PMC4978255

[ref16] Cohen J. (1992). A power primer.Pdf. In Psychological Bulletin 1, 155–159. 10.1037//0033-2909.112.1.155 19565683

[ref17] Confucius. (1980). The analects of Confucius. China: Zhonghua Book Company.

[ref18] Cooper J. M. Hutchinson D. S. (1997). Plato: Complete works. United States: Hackett Publishing.

[ref19] Cronin A. L. (2014). Ratio-dependent quantity discrimination in quorum sensing ants. Anim. Cogn. 17, 1261–1268. doi: 10.1007/s10071-014-0758-8, PMID: 24844665

[ref20] DeLeeuw J. L. Galen L. W. Aebersold C. Stanton V. (2007). Support for animal rights as a function of belief in evolution, religious fundamentalism, and religious denomination. Soc. Anim. 15, 353–363. doi: 10.1163/156853007X235528

[ref21] Dweck C. S. (1999). Self-theories: Their role in motivation, personality, and development. London: Psychology press. 2130257

[ref22] Farroni T. Johnson M. H. Menon E. Zulian L. Faraguna D. Csibra G. (2005). Newborns’ preference for face-relevant stimuli: effects of contrast polarity. Proc. Natl. Acad. Sci. 102, 17245–17250. doi: 10.1073/pnas.0502205102, PMID: 16284255 PMC1287965

[ref23] Feigenson L. Carey S. Hauser M. (2002). The representations underlying infants’ choice of more: object files versus analog magnitudes. Psychol. Sci. 13, 150–156. doi: 10.1111/1467-9280.00427, PMID: 11933999

[ref24] Fodor J. A. (1983). The modularity of mind. United States: MIT press.

[ref25] Gopnik A. Meltzoff A. N. (1997). Words, thoughts, and theories. United States: MIT Press.

[ref26] Gross H. J. Pahl M. Si A. Zhu H. Tautz J. Zhang S. (2009). Number-based visual generalisation in the honeybee. PLoS One 4:e4263. doi: 10.1371/journal.pone.0004263, PMID: 19173008 PMC2629729

[ref27] Hamlin J. K. Wynn K. Bloom P. (2007). Social evaluation by preverbal infants. Nature 450, 557–559. doi: 10.1038/nature06288, PMID: 18033298

[ref28] Hasbrouck J. Tindal G. A. (2006). Oral Reading fluency norms: a valuable assessment tool for Reading teachers. Read. Teach. 59, 636–644. doi: 10.1598/RT.59.7.3

[ref29] Izard V. Sann C. Spelke E. S. Streri A. (2009). Newborn infants perceive abstract numbers. Proc. Natl. Acad. Sci. 106, 10382–10385. doi: 10.1073/pnas.0812142106, PMID: 19520833 PMC2700913

[ref30] Johnson S. Slaughter V. Carey S. (1998). Whose gaze will infants follow? The elicitation of gaze-following in 12-month-olds. Dev. Sci. 1, 233–238. doi: 10.1111/1467-7687.00036

[ref31] Lakens D. Scheel A. M. Isager P. M. (2018). Equivalence testing for psychological Research: a tutorial. Adv. Methods Pract. Psychol. Sci. 1, 259–269. doi: 10.1177/2515245918770963

[ref32] Landau B. Spelke E. (1988). Geometric complexity and object search in infancy. Dev. Psychol. 24, 512–521. doi: 10.1037/0012-1649.24.4.512

[ref33] Locke J. (1847). An essay concerning human understanding, Philadelphia: Kay & Troutman.

[ref34] Meng X. Nakawake Y. Hashiya K. Burdett E. Jong J. Whitehouse H. (2021). Preverbal infants expect agents exhibiting counterintuitive capacities to gain access to contested resources. Sci. Rep. 11:10884. doi: 10.1038/s41598-021-89821-0, PMID: 34035341 PMC8149634

[ref35] Muller M. Wehner R. (1988). Path integration in desert ants, Cataglyphis fortis. Proc. Natl. Acad. Sci. 85, 5287–5290. doi: 10.1073/pnas.85.14.5287, PMID: 16593958 PMC281735

[ref36] Nagata T. Koyanagi M. Tsukamoto H. Saeki S. Isono K. Shichida Y.. (2012). Depth perception from image defocus in a jumping spider. Science 335, 469–471. doi: 10.1126/science.1211667, PMID: 22282813

[ref37] Norenzayan A. Gervais W. M. (2013). The origins of religious disbelief. Trends in Cognitive Sciences 17, 20–25. doi: 10.1016/j.tics.2012.11.006 23246230

[ref38] Northern J. L. Downs M. P. (2002). Hearing in children. United States: Lippincott Williams & Wilkins.

[ref39] Piaget J. (1954). The construction of reality in the child. Basic Books. doi: 10.1037/11168-000

[ref40] Piaget J. Inhelder B. (2008). The psychology of the child. United States: Basic books.

[ref41] Piffer L. Agrillo C. Hyde D. C. (2012). Small and large number discrimination in guppies. Anim. Cogn. 15, 215–221. doi: 10.1007/s10071-011-0447-9, PMID: 21909934

[ref42] Pinker S. (2004). Why nature & nurture won’t go away. Daedalus 133, 5–17. doi: 10.1162/0011526042365591

[ref43] Przyrembel C. Keller B. Neumeyer C. (1995). Trichromatic color vision in the salamander (Salamandra salamandra). J. Comp. Physiol. A. 176, 575–586. doi: 10.1007/BF00196422

[ref44] Rogers J. L. Howard K. I. Vessey J. T. (1993). Using significance tests to evaluate equivalence between two experimental groups. Psychol. Bull. 113, 553–565. doi: 10.1037/0033-2909.113.3.553, PMID: 8316613

[ref45] Rosa-Salva O. Regolin L. Vallortigara G. (2009). Faces are special for newly hatched chicks: evidence for inborn domain-specific mechanisms underlying spontaneous preferences for face-like stimuli. Dev. Sci. 13, 565–577. doi: 10.1111/j.1467-7687.2009.00914.x, PMID: 20590721

[ref46] Rosenthal R. (1969). Task variations in studies of experimenter expectancy effects. Percept. Mot. Skills 29, 9–10. doi: 10.2466/pms.1969.29.1.9

[ref47] Saffran J. R. Aslin R. N. Newport E. L. (1996). Statistical learning by 8-month-old infants. Science 274, 1926–1928. doi: 10.1126/science.274.5294.1926 8943209

[ref48] Schloegl C. Kotrschal K. Bugnyar T. (2007). Gaze following in common ravens, Corvus corax: ontogeny and habituation. Anim. Behav. 74, 769–778. doi: 10.1016/j.anbehav.2006.08.017

[ref49] Skinner B. F. (1948). ‘Superstition’in the pigeon. J. Exp. Psychol. 38, 168–172. doi: 10.1037/h0055873 18913665

[ref50] Slater A. Mattock A. Brown E. (1990). Size constancy at birth: newborn infants’ responses to retinal and real size. J. Exp. Child Psychol. 49, 314–322. doi: 10.1016/0022-0965(90)90061-C, PMID: 2332727

[ref51] Spelke E. S. Kinzler K. D. (2007). Core knowledge. Dev. Sci. 10, 89–96. doi: 10.1111/j.1467-7687.2007.00569.x 17181705

[ref52] Stahl A. E. Feigenson L. (2015). Observing the unexpected enhances infants’ learning and exploration. Science 348, 91–94. doi: 10.1126/science.aaa3799, PMID: 25838378 PMC5861377

[ref53] Stankov L. (2010). Unforgiving Confucian culture: a breeding ground for high academic achievement, test anxiety and self-doubt? Learn. Individ. Differ. 20, 555–563. doi: 10.1016/j.lindif.2010.05.003

[ref54] Starkey P. Cooper R. G. (1980). Perception of numbers by human infants. Science 210, 1033–1035. doi: 10.1126/science.7434014 7434014

[ref55] Stevenson H. W. Lee S.-Y. Chen C. Stigler J. W. Hsu C.-C. Kitamura S.. (1990). Contexts of achievement: a study of American, Chinese, and Japanese children. Monogr. Soc. Res. Child Dev. 55, 1–119. doi: 10.2307/1166090 2342493

[ref56] Sun X. Nancekivell S. Gelman S. A. Shah P. (2020). Perceptions of the malleability of fluid and crystallized intelligence. J. Exp. Psychol. Gen. 150, 815–827. doi: 10.1037/xge0000980, PMID: 33001687

[ref57] The Ministry of Education, Culture, Sports, Science and Technology, M (2017). 子供の読書活動に関する現状と論点 (Vol. 4,). Available at: https://www.mext.go.jp/b_menu/shingi/chousa/shougai/040/shiryo/__icsFiles/afieldfile/2017/08/15/1389071_005.pdf (Accessed July 31, 2022).

[ref58] Thomsen L. Frankenhuis W. E. Ingold-Smith M. Carey S. (2011). Big and mighty: preverbal infants mentally represent social dominance. Science 331, 477–480. doi: 10.1126/science.1199198, PMID: 21273490 PMC3860821

[ref59] Vallortigara G. Regolin L. Rigoni M. Zanforlin M. (1998). Delayed search for a concealed imprinted object in the domestic chick. Anim. Cogn. 1, 17–24. doi: 10.1007/s100710050003

[ref60] Wang J. J. Feigenson L. (2019). Is empiricism innate? Preference for nurture over nature in People’s beliefs about the origins of human knowledge. Open Mind 3, 89–100. doi: 10.1162/opmi_a_00028, PMID: 34485789 PMC8412204

