# Supplementary material for: A cross-cultural investigation of people’s intuitive beliefs about the origins of cognition
Source: Front Psychol. 2022 Nov 8;13:974434. doi: 10.3389/fpsyg.2022.974434 (PMC9683109; doi:10.3389/fpsyg.2022.974434)
Supplement: Supplementary file 1 [file Data_Sheet_1.pdf]

Table S1. Survey questions in Study 1A and Study 2.

Ability	Age Onset Question	Ability Origin Question	Age Onset Question (In Japanese)	Ability Origin Question (In Japanese)	Study 1A	Study 2
**See**	Alex can see things with her eyes. When could Alex see with her eyes for the first time?	How come she can see?	ヒカルさんは目で物を見ることができます。初めてヒカルさんが目で物を見ることができたのはいつだと思いますか？	なぜ、ヒカルさんは見ることができると思いますか？	●	●
**Hear**	When there is a sound close by, Alex can hear it. When could Alex hear sounds for the first time?	How come she can hear?	すぐそばで音がしたときにヒカルさんはそれを聞くことができます。初めてヒカルさんが音を聞くことができたのはいつだと思いますか？	なぜ、ヒカルさんは音を聞くことができると思いますか？	●	●
**Discriminate colors**	When seeing a red flower and a blue flower, Alex can tell that they are different colors. Alex can tell colors apart. When could Alex tell colors apart for the first time?	How come she can tell colors apart?	赤いりんごと青いりんごを見たとき、ヒカルさんはそれらが違う色だとわかります。ヒカルさんは色の違いを見分けることができます。ヒカルさんが初めて色の違いを見分けることができたのはいつだと思いますか？	なぜ、ヒカルさんは色の違いを見分けることができると思いますか？	●	●
**Discriminate distances**	When there is a car approaching, Alex can tell that the car is getting closer. Alex can tell what is near and what is far. When could Alex tell near and far for the first time?	How come she can tell near and far?	ヒカルさんは、車が近くに来た時にその車が接近してきたことがわかります。ヒカルさんは、遠くと近くを見分けることができます。初めてヒカルさんが遠近がわかったのはいつだと思いますか？	なぜ、ヒカルさんは遠近がわかると思いますか？	●	●
**Prefer faces to non-faces**	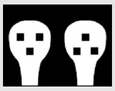 If Alex sees the above pictures, Alex thinks that the picture on the left looks a bit more like a face. Alex can tell whether something looks like a face or not. When was the first time Alex could tell whether something looks like a face?	How come she can tell whether something looks like a face?	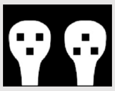 ヒカルさんが上の画像を見ると、左側の絵が少し顔のように見えると思います。ヒカルさんは何かが顔に見えるかどうかを見分けることができます。ヒカルさんが顔のように見えるものを初めて見分けることができたのはいつだと思いますか？	なぜ、ヒカルさんは何かが顔のように見えるかを見分けることができると思いますか？	●	●
**Think unsupported objects will fall**	When Alex sees someone hold an object and then drop it, Alex thinks the object will fall. Alex thinks objects will fall if we let go of them. When could Alex think that for the first time?	How come she can think that objects will fall if we let go of them?	ヒカルさんは、ある人が物を落とすところを見ると、その物は落下するだろうと思います。ヒカルさんは物を手放すとそれは落ちると思うのです。初めてヒカルさんがそう思ったのはいつだと思いますか？	なぜ、ヒカルさんは物を手放すとそれは落下すると思うのでしょうか？	●	●
**Think hidden objects are still there**	If Alex sees a toy being hidden in a box, she will think the object is still there even though she can no longer see it. When could Alex think that for the first time?	How come she can think that hidden objects will still be there?	ヒカルさんは、おもちゃが箱の中に隠されているところを見たとすると、もうおもちゃが見えなくても、まだそれが箱の中にあると思います。初めてヒカルさんがそのように思ったのはいつだと思いますか？	なぜ、ヒカルさんは隠されたものがまだそこにあると思うのでしょうか？	●	●
**Discriminate quantities**	If Alex sees two cookies, one with 5 chocolate chips in it and one with 20 chocolate chips in it, she can tell which cookie has more chocolate chips without counting. Alex can tell which has more. When could Alex tell which has more for the first time?	How come she can tell which has more?	1枚に5個のチョコチップが、もう1枚には20個のチョコチップが入っているクッキーをヒカルさんが見たとします。彼女は数えなくてもどちらのクッキーのチョコチップが多いかが分かります。ヒカルさんはどちらが多いかを見分けることができます。彼女が初めてどちらが多いか見分けることができたのはいつだと思いますか？	なぜ、ヒカルさんはどちらの方が多いか見分けることができると思いますか？	●	●
**Prefer helping to not helping**	If Alex sees a turtle that is upside down and struggling to get on its feet, she thinks that she should help the turtle. Alex thinks that helping is the right thing to do. When could Alex think that for the first time?	How come she can think that helping is right?	ひっくり返って足をばたつかせているカメを見たヒカルさんは、カメを助けるべきだと思います。ヒカルさんはカメを助けることが正しいことだと思っています。ヒカルさんが初めてそう思ったのはいつだと思いますか？	なぜ、ヒカルさんは助けることが正しいと思うのでしょうか？	●	●
**Read**	Alex can read books. When could Alex read for the first time?	How come she can read?	ヒカルさんは本を読むことができます。初めてヒカルさんが本を読むことができたのはいつだと思いますか？	なぜ、ヒカルさんは本を読むことができると思いますか？	●	●
**Think bigger is mightier**	When Alex sees a physically bigger character fighting with a smaller character, she thinks that the bigger character will win. Alex thinks that bigger characters will win fights. When was the first time Alex thinks that bigger character wins fights?	How come she can think that bigger characters will win fights?	ヒカルさんは体が大きいキャラクターと小さいキャラクターが戦っている場面を見たとします。彼女は大きいほうが勝つだろうと思います。ヒカルさんは、体が大きいほうが勝負に勝つと予測できるのです。初めてヒカルさんが体が大きいほうが勝負に勝つと予測できたのはいつだと思いますか？	なぜ、ヒカルさんは体が大きいほうが勝負に勝つと予測できると思いますか？		●

**Explore unexpected events**	When Alex sees surprising events such like a toy car pass through a solid wall, Alex will want to figure out how it happened. When Alex sees something surprising, she wants to figure out how it happened. When could Alex first want to know the reason behind surprising events?	How come she can want to know the reason behind surprising events?	ヒカルさんがおもちゃの車が固そうな壁を通り抜けるというような、不思議な場面を見たとします。彼女はその後、車や壁を触ってなぜ車が通り抜けられたかを知ろうとします。ヒカルさんは、不思議な現象を見たときにその原因を知ろうとするのです。初めてヒカルさんが不思議なことの原因を知ろうしたのはいつだと思いますか？	なぜ、ヒカルさんは不思議な現象の原因を知ろうとすると思いますか？		•
**Learn from patterns**	After seeing or hearing things together in a pattern many times (such as hearing “pretty baby”), Alex can recognize the familiar pattern. Alex can tell familiar patterns from new ones based on what she's heard or seen. When could Alex recognize familiar patterns based on what she's heard or seen for the first time?	How come she can recognize familiar patterns based on experience?	ヒカルさんがあるパターンを繰り返して見たり聞いたりすると（例えば「かわいい　赤ちゃん」を何回も聞く）、彼女はそのパターンがなじみのパターンだと認識することができます。ヒカルさんは、見たものや聞いたものについて、新しいパターンから、なじみのパターンを見分けることができます。はじめてヒカルさんがなじみのパターンを認識できるのはいつだと思いますか。	なぜ、ヒカルさんはパターンを認識することができると思いますか？		•

Table S2. Survey questions in Study 1B.

**Ability**	**Age Onset Question**	**Ability Origin Question**	**Age Onset Question (In Japanese)**	**Ability Origin Question (In Japanese)**
**See**	Alex can see things with her eyes. When could Alex see with her eyes for the first time?	How did Alex become able to see?	ヒカルさんは目で物を見ることができます。初めてヒカルさんが目で物を見ることができたのはいつだと思いますか？	なぜ、ヒカルさんは物を見ることができると思いますか？
**Hear**	When there is a sound close by, Alex can hear it. When could Alex hear sounds for the first time?	How did Alex become able to hear?	すぐそばで音がしたときにヒカルさんはそれを聞くことができます。初めてヒカルさんが音を聞くことができたのはいつだと思いますか？	なぜ、ヒカルさんは音を聞くことができたと思いますか？
**Discriminate colors**	When seeing a red flower and a blue flower, Alex can see that they have different colors. Alex can see the difference between colors. When could Alex see the difference between colors for the first time?	How did Alex come to see the difference between colors?	赤い花と青い花を見たとき、ヒカルさんはそれらが違う色だということがわかります。ヒカルさんは色の違いがわかります。ヒカルさんが初めて色の違いがわかったのはいつだと思いますか？	なぜ、ヒカルさんは色の違いがわかると思いますか？
**Discriminate distances**	When there is an object approaching, Alex can reach for the object when the object gets closer. Alex reaches for close-by things rather than far-away things. When could Alex first reach more for close-by things than far-away things?	How did Alex come to reach more for close-by things than far-away things?	ある物が近くにくると、ヒカルさんはその物に手を伸ばすことができます。ある物が遠くにある場合より、近くにある方がヒカルさんはより頻繁に手を伸ばします。ヒカルさんが初めて近くのものにより頻繁に手を伸ばすようになったのはいつだと思いますか？	なぜ、ヒカルさんは近くのものにより頻繁に手を伸ばすようになったと思いますか？
**Prefer faces to non-faces**	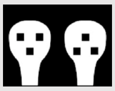 If Alex sees the above pictures, Alex looks longer at the picture on the left. Alex prefers to look at face- like shapes than at other shapes. When was the first time Alex preferred to look at face-like shapes over other shapes?	How did Alex come to prefer face-like shapes over other shapes?	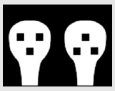 ヒカルさんが上の画像を見ると、彼女は左側の絵の方をより長く見ます。ヒカルさんは他の形よりも顔のような形を見る方を好みます。ヒカルさんが他の形よりも顔のような形を好んで見たのはいつだと思いますか？	なぜ、ヒカルさんは他の形よりも顔のような形を好んで見たと思いますか？
**Think unsupported objects will fall**	When Alex sees someone hold an object and then drop it, Alex reaches out for the object because it is about to fall. Alex can reach for things that are about to fall. When could Alex first reach out for an object that is about to fall?	How did Alex come to reach out for an object that's about to fall?	誰かが手に持っている物が落ちそうになるのを見たとき、ヒカルさんはそれを受け止めようと手を伸ばします。ヒカルさんは落ちそうな物に手を伸ばすことができます。初めてヒカルさんが落ちそうなものに手を伸ばすことができたのはいつだと思いますか？	なぜ、ヒカルさんは落ちそうな物に手を伸ばして受け止めることができたと思いますか？
**Think hidden objects are still there**	If Alex sees a toy being hidden in a box, she reaches for the toy even though she can no longer see it. When could she first reach for a toy that she could no longer see?	How did Alex come to reach for a hidden object?	おもちゃが箱の中に隠されているところを見たヒカルさんは、もうそれが見えなくても、おもちゃに手を伸ばします。初めてヒカルさんが隠されて見ることができないおもちゃに手を伸ばすことができたのはいつだと思いますか?	なぜ、ヒカルさんは隠された物に手を伸ばしたと思いますか？

**Discriminate quantities**	If Alex sees two cookies, one with 5 chocolate chips in it and one with 20 chocolate chips in it, she reaches for the cookie with more chocolate chips, without counting. Alex reaches for the amount that's more. When could she reach for something with more for the first time?	How did Alex come to be able to reach for things with more?	1枚に5個のチョコチップが、もう1枚には20個のチョコチップが入っているクッキーをヒカルさんが見るとすると、彼女は数えなくてもチョコチップの多いクッキーに手を伸ばします。ヒカルさんは、数がより多い方を取ろうとします。彼女が初めて数がより多い方を取ろうとしたのはいつだと思いますか？	なぜ、ヒカルさんは数がより多い方に手を伸ばすことができたと思いますか？
**Prefer helping to not helping**	Alex sees a turtle that is upside down and struggling to get on its feet. If she then sees one person help the turtle get back on its feet, and another person let the turtle continue to struggle, Alex prefers the person who helped over the person who did not help. When was the first time Alex preferred helping?	How did Alex come to prefer helping?	ヒカルさんは裏返ってもがいているカメを見ました。もし、ある人が亀を助けて立ち直らせ、別の人はカメがもがいていてもそのままにしているのを彼女が見るとすると、彼女は亀を助けなかった人より助けた人の方が好ましいと思います。初めてヒカルさんが助ける方が好ましいと思ったのはいつだと思いますか？	なぜ、ヒカルさんは助ける方が好ましいと思うのでしょうか？
**Read**	Alex can read books. When could Alex read for the first time?	How did Alex become able to read?	ヒカルさんは本を読むことができます。初めてヒカルさんが本を読むことができたのはいつだと思いますか？	なぜ、ヒカルさんは本を読むことができるようになったと思いますか？

Table S3. Survey questions in Study 1C - Human Condition.

Ability	Ability Origin Question	Age Onset Question	Ability Origin Question (In Japanese)	Age Onset Question (In Japanese)
**See**	Alex can see things with her eyes. How come she can see?	Can a baby that was just born see?	ヒカルさんは目で物を見ることができます。なぜ、ヒカルさんは見ることができると思いますか？	生まれたばかりの赤ちゃんは、見ることができると思いますか？
**Prefer faces to non-faces**	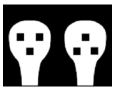 If Alex sees the above pictures, Alex can tell that the picture on the left looks a bit more like a face. Alex can tell whether something looks like a face or not. How come Alex can tell whether something looks like a face?	Can a baby that was just born tell whether something looks like a face?	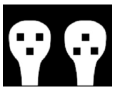 ヒカルさんが上の画像を見ると、左側の絵は少し顔のように見えます。ヒカルさんは何かが顔のように見えるかどうかを見分けることができます。なぜ、ヒカルさんは何かが顔のように見えるかを見分けることができると思いますか？	生まれたばかりの赤ちゃんは、何かが顔のように見えるかを見分けることができると思いますか？
**Discriminate distances**	When there is a car approaching, Alex can tell that the car is getting closer. Alex can tell what is near and what is far. How come Alex can tell near and far?	Can a baby that was just born tell near and far?	ヒカルさんは車が近くに来たとき、その車が接近してきていることがわかります。ヒカルさんは遠くと近くがわかるのです。なぜ、ヒカルさんは遠近を見分けることができると思いますか？	生まれたばかりの赤ちゃんは、遠近を見分けることができると思いますか？
**Discriminate colors**	When seeing a red flower and a blue flower, Alex can tell that they are different colors. Alex can tell colors apart. How come Alex can tell colors apart?	Can a baby that was just born tell colors apart?	赤い花と青い花を見たとき、ヒカルさんはそれらは違う色だとわかります。ヒカルさんは色の違いがわかるのです。なぜ、ヒカルさんは色の違いがわかると思いますか？	生まれたばかりの赤ちゃんは、色の違いがわかると思いますか？
**Perceive angle of body rotation**	If Alex sits on a spinning chair and someone turns the chair by 90 degrees, Alex can tell that her body was turned by about 90 degrees, even if there are no visual cues available. Alex can tell approximately how much her body turns. How come Alex can tell approximately how much her body turns?	If someone holds a baby and turns it gently, can a baby that was just born tell approximately how much its body turns?	ヒカルさんが回転する椅子に座っていて、誰かがその椅子を90度回転させたとします。たとえ視覚的な手がかりがなくても、ヒカルさんは自分の体がだいたい90度回転したことがわかります。ヒカルさんは自分の体がおおよそどれだけ回転したか分かるのです。ヒカルさんは、なぜ自分の体がだいたいどれだけ回転したかわかるのですか？	生まれたばかりの赤ちゃんが抱っこされた状態で回転させられたとしたら、その赤ちゃんは自分の体がおおよそどのくらい回転したかわかると思いますか？
**Follow the gaze of others**	When Alex sees someone looking at something, Alex will often follow their gaze to see what they are looking at. Alex can follow the gaze of others to see what they are looking at. How come Alex can follow the gaze of others to see what they are looking at?	Can a baby that was just born follow the gaze of others to see what they are looking at?	ヒカルさんは、誰かが何かを見ているのを見ると、その視線を追うことで何を見ているかが分かることがよくあります。ヒカルさんは他人の視線を追うことができ、その人が何を見ているかを知ることができます。ヒカルさんはなぜ他人の視線を追うことで何を見ているかを知ることができるのでしょうか？	生まれたばかりの赤ちゃんは、他人の視線を追って何を見ているかを知ることができると思いますか？

**Discriminate two things from three**	If Alex sees an image with 2 dots and an image with 3 dots, Alex can tell them apart. Alex can tell the difference between 2 things and 3 things. How come Alex can tell the difference between 2 things and 3 things?	Can a baby that was just born tell the difference between 2 things and 3 things?	ヒカルさんが、一枚の写真には２個のドット、もう一枚の写真には３個のドットがある、二枚の写真を見ているとします。彼女はこの二枚の写真の２個と３個のドットの違いが分かります。なぜ、ヒカルさんは２個と３個の違いを見分けることができると思いますか？	生まれたばかりの赤ちゃんは、２個と３個の違いを見分けることができると思いますか？
**Discriminate quantities**	If Alex sees two cookies, one with 5 chocolate chips in it and one with 20 chocolate chips in it, she can tell which cookie has more chocolate chips without counting. Alex can tell which is more. How come Alex can tell which is more?	Can a baby that was just born tell which is more?	一枚に5個のチョコチップが入っていて、もう一枚には20個のチョコチップが入っている二枚のクッキーをヒカルさんが見たとします。ヒカルさんはどちらのクッキーの方がチョコチップが多いかを数えなくても分かります。彼女はどちらが多いかを見分けることができるのです。なぜ、彼女は多い方を見分けることができるのでしょう？	生まれたばかりの赤ちゃんは、どちらが多いかを見分けることができますか？
**Think hidden objects are still there**	If Alex sees a toy being hidden in a box, she can tell that the object is still there even though she can no longer see it. How come Alex can tell that a hidden object is still there even though she can't see it?	Can a baby that was just born tell that an object is still there even when it can't be seen (e.g., if it is hidden behind a larger object)?	ヒカルさんは、箱の中におもちゃが隠されているところを見ると、たとえおもちゃが見えなくても、それが箱の中にあることが分かります。なぜヒカルさんは、見えなくても隠された物がまだそこにあると分かるのですか。	生まれたばかりの赤ちゃんは、隠れて見えなくても、それがまだそこにあることがわかると思いますか？（例えば，ある物体がより大きな物体の後ろに隠れた場合）
**Wash hands/Use litterbox**	Alex usually washes hands before she eats. Alex thinks that people should wash her hands before eating. How come Alex can think that people should wash hands before eating?	Can a baby that was just born think that people should wash hands before eating?	ヒカルさんは普通食事の前に手を洗います。ヒカルさんは食事の前に手を洗うべきだと思います。なぜ、ヒカルさんは食事の前に手を洗うべきだと思えるのでしょうか？	生まれたばかりの赤ちゃんは、食事の前に手を洗うべきだと思うことができますか？

Table S4. Survey questions in Study 1C - Animal Condition.

Ability	Ability Origin Question	Age Onset Question	Ability Origin Question (In Japanese)	Age Onset Question (In Japanese)
**See**	A horse can see with its eyes. How come a horse can see?	Can a baby horse that was just born see?	馬は目で見ることができます。なぜ、馬は見ることができると思いますか？	生まれたばかりの馬の赤ちゃんは、見ることができると思いますか？
**Prefer faces to non-faces**	A chicken can tell whether something looks like a chicken or not. How come a chicken can tell whether something looks like a chicken?	Can a baby chicken that just hatched tell whether something looks like a chicken?	ニワトリは、何かがニワトリに似ているかどうかがわかります。なぜ、ニワトリは何かがニワトリに似ているかどうかがわかると思いますか？	生まれたばかりのニワトリの赤ちゃんは、何かがニワトリに似ているかそうでないかがわかると思いますか？
**Discriminate distances**	When there is a bug flying by, a spider can tell that the bug is getting closer. A spider can tell what is near and what is far. How come a spider can tell near and far?	Can a baby spider that just hatched tell near and far?	虫が飛んで来ると、クモは虫が近づいていることがわかります。クモは近くに何があり、遠くに何があるかが分かります。なぜ、クモは遠近がわかると思いますか?	孵化したばかりのクモの赤ちゃんは、遠近がわかると思いますか？
**Discriminate colors**	When seeing a blue insect and a red insect, a salamander can tell that they are different colors. A salamander can tell colors apart. How come a salamander can tell colors apart?	Can a baby salamander that just hatched tell colors apart?	サラマンダー（トカゲ）は青い虫と赤い虫を見ると色が違うことがわかります。サラマンダーは色の違いを見分けることができます。なぜ、サラマンダーは色の違いが分かると思いますか?	生まれたばかりのサラマンダーの赤ちゃんは、色の違いがわかると思いますか？
**Perceive angle of body rotation**	An ant that is foraging for food can tell how much its body turns as it walks around, even if there are no visual cues available. An ant can tell approximately how much its body turns as it moves. How come an ant can tell approximately how much its body turns as it moves?	Can a baby ant that just hatched tell approximately how much its body turns (once it can move)?	餌を探しているアリは、視覚的な手がかりがなくても、歩き回りながら体がどれだけ回転するかがわかります。アリは動きながらだいたいどのくらい体が回転するかがわかるのです。なぜアリは体がどのくらい回転するかがわかるのでしょうか？	生まれたばかりのアリの赤ちゃんは（動けるようになってから）、体がだいたいどれくらい回転するかわかると思いますか？
**Follow the gaze of others**	When a crow sees someone looking at something, it often will follow their gaze to see what they are looking at. A crow can follow the gaze of others to see what they are looking at. How come a crow can follow the gaze of others to see what they are looking at?	Can a baby crow that was just born follow the gaze of others to see what they are looking at?	カラスは誰かが何かを見ているのを見ると、それらの視線を追って何を見ているのかを知ることがよくあります。カラスは他の個体の視線を追うことで、それらが何を見ているのかを知ることができます。なぜ、カラスは他の個体の視線を追うことができると思いますか？	生まれたばかりのカラスの赤ちゃんは、他の個体の視線を追って何を見ているのかを知ることができると思いますか？

**Discriminate two things from three**	If a bee sees an image with 2 things and an image with 3 things, the bee can tell them apart. A bee can tell the difference between 2 things and 3 things. How come a bee can tell the difference between 2 things and 3 things?	Can a baby bee that just hatched tell the difference between 2 things and 3 things?	一枚の写真には２個のものがあり、もう一枚の写真には３個のものがある、二枚の写真をハチが見ているとすると、ハチはこの二枚の写真の違いがわかります。ハチは２個と３個の違いがわかるのです。なぜ、ハチは２個と３個の違いがわかると思いますか？	生まれたばかりのハチの赤ちゃんは、２個と３個の違いがわかると思いますか？
**Discriminate quantities**	If a fish sees two groups of fish, one group with 5 fish and one with 20 fish, the fish can tell which group has more fish. The fish can tell which is more. How come a fish can tell which is more?	Can a baby fish that was just born tell which is more?	ある魚が二つの魚グループを見ているとします。一方のグループには５匹の魚がいて、もう一方には２０匹の魚がいる場合、この魚はどちらのグループの魚が多いかを見分けることができます。魚はどちらの方がより多いかがわかるのです。なぜ、魚はどちらの方が多いかがわかると思いますか？	生まれたばかりの魚の赤ちゃんは、どちらの方が多いかがわかると思いますか？
**Think hidden objects are still there**	When a chicken sees an object being hidden behind a wall, the chicken can tell that the object is still there even though it cannot see it. How come a chicken can tell that an object is still there even when it cannot see the object (for example, if the object is hidden behind a wall)?	Can a baby chicken that just hatched tell that a hidden object is still there even though it cannot see it?	ニワトリは、壁の後ろに隠れている物体を見ると、たとえそれが見えなくてもその物体がまだそこにあることがわかります。なぜ、ニワトリは物体を見なくても、その物体がまだそこにあることがわかるのでしょうか？(たとえば、物体が壁の背後に隠れている場合)	生まれたばかりのニワトリの赤ちゃんは、たとえそれが見えなくても隠れている物体があることがわかると思いますか？
**Wash hands/Use litterbox**	A house-cat uses a litterbox to go to the bathroom. When it has to go to the bathroom, it can find its litterbox and use it to go to the bathroom. How come the cat can use the litterbox to go to the bathroom?	Can a baby kitten that was just born use the litterbox to go to the bathroom?	家猫はトイレに行くのに猫トイレを使います。トイレに行くときは猫トイレを見つけて、それを使ってトイレをすることができます。なぜ、家猫は猫トイレを使うことができると思いますか？	生まれたばかりの猫の赤ちゃんは、猫トイレが使えると思いますか？

Table S5. Results of Study 1A.

	JP									
	Estimated age onset					Frequency of explanations				
Ability	*Mean*	*SD*	*Position on the picture scale representing empirical ages*	*T (Compared to the max age of the position)*	*P-value*	*Born able to X*	*X matured with age*	*Learned X on own*	*Taught X by others*	*Uncategorized*
See	0.42	0.23	(≦ 0.5)	-3.57	< .001	50	13	3	0	34
Hear	0.42	0.35	(≦ 0.5)	-2.40	0.018	55	16	1	0	28
Discriminate colors	1.36	1.31	(≦ 0.5)	6.51	< .001	18	14	16	11	41
Discriminate distances	2.60	2.60	(≦ 0.5)	8.10	< .001	7	14	29	7	43
Prefer faces to non-faces	1.61	1.65	(≦ 0.5)	6.73	< .001	7	7	40	1	45
Think unsupported objects will fall	2.57	3.09	(≦ 0.5)	6.69	< .001	0	1	81	3	15
Think hidden objects are still there	1.98	1.80	(≦ 0.5)	8.24	< .001	1	8	41	1	49
Discriminate quantities	2.98	2.55	(≦ 0.5)	9.72	< .001	4	12	25	12	47
Prefer helping to not helping	4.25	3.19	(≦ 0.5)	11.74	< .001	2	5	29	29	35
Read	3.43	1.76	(4 - 7)	-20.30	< .001	0	2	20	61	17

	US									
	Estimated age onset					Frequency of explanations				
Ability	*Mean*	*SD*	*Position on the picture scale representing empirical ages*	*T (Compared to the max age of the position)*	*P-value*	*Born able to X*	*X matured with age*	*Learned X on own*	*Taught X by others*	*Uncategorized*
See	0.44	1.37	(≦ 0.5)	-0.43	0.668	88	5	1	0	6
Hear	0.36	0.31	(≦ 0.5)	-4.53	< .001	91	2	2	0	5
Discriminate colors	1.69	1.31	(≦ 0.5)	9.08	< .001	23	12	26	29	10
Discriminate distances	2.53	2.19	(≦ 0.5)	9.28	< .001	13	12	44	12	19
Prefer faces to non-faces	1.33	1.28	(≦ 0.5)	6.47	< .001	22	7	48	4	19
Think unsupported objects will fall	1.51	1.13	(≦ 0.5)	8.92	< .001	2	2	78	4	14
Think hidden objects are still there	1.93	1.28	(≦ 0.5)	11.19	< .001	1	5	58	4	32
Discriminate quantities	3.04	1.98	(≦ 0.5)	12.82	< .001	6	3	38	28	25
Prefer helping to not helping	3.49	2.02	(≦ 0.5)	14.83	< .001	7	3	28	38	24
Read	5.08	1.88	(4 - 7)	-10.18	< .001	0	0	12	85	3

Table S6. Results of Study 1B.

	Estimated age onset					Frequency of explanations				
Ability	*Mean*	*SD*	*Position on the picture scale representing empirical ages*	*T (Compared to the max age of the position)*	*P-value*	*Born able to X*	*X matured with age*	*Learned X on own*	*Taught X by others*	*Uncategorized*
See	0.39	0.16	(≦ 0.5)	-7.23	< .001	44	22	0	0	34
Hear	0.35	0.16	(≦ 0.5)	-9.20	< .001	61	17	1	0	21
Discriminate colors	1.21	0.90	(≦ 0.5)	7.87	< .001	27	20	3	7	43
Discriminate distances	1.39	2.27	(≦ 0.5)	3.94	< .001	8	36	14	0	42
Prefer faces to non-faces	1.22	1.85	(≦ 0.5)	3.90	< .001	6	16	6	0	72
Think unsupported objects will fall	3.79	3.74	(≦ 0.5)	8.81	< .001	8	36	24	0	32
Think hidden objects are still there	1.64	1.03	(≦ 0.5)	11.06	< .001	1	26	27	0	46
			(0.5 - 1)*	6.19	< .001	*Piaget (1954)				
Discriminate quantities	3.29	2.63	(≦ 0.5)	10.64	< .001	2	30	21	4	43
			(0.5 - 1)*	8.74	< .001	*Feigenson et al (2002)				
Prefer helping to not helping	5.32	5.06	(≦ 0.5)	9.52	< .001	2	31	20	15	32
Read	3.27	2.17	(4 - 7)	-17.16	< .001	0	16	9	60	15

Table S7. Results of Study 1C.

Human Condition	Percentage endorsing nature		Frequency of explanations				
Ability	*Mean*	*SD*	*Born able to X*	*X matured with age*	*Learned X on own*	*Taught X by others*	*Uncategorized*
See	0.79	0.41	48	9	4	0	39
Prefer faces to non-faces	0.47	0.50	15	5	31	0	49
Discriminate distances	0.35	0.48	25	6	32	3	34
Discriminate colors	0.69	0.47	31	3	15	6	45
Perceive angle of body rotation	0.31	0.47	13	5	33	0	49
Follow the gaze of others	0.35	0.48	16	10	29	4	41
Discriminate two things from three	0.33	0.47	12	6	16	13	53
Discriminate quantities	0.20	0.40	9	7	23	8	53
Think hidden objects are still there	0.16	0.37	6	6	57	4	27
Wash hands/Use litterbox	0.02	0.14	1	2	15	70	12

Animal Condition	Percentage endorsing nature		Frequency of explanations				
Ability	*Mean*	*SD*	*Born able to X*	*X matured with age*	*Learned X on own*	*Taught X by others*	*Uncategorized*
See	0.86	0.35	47	1	0	0	52
Prefer faces to non-faces	0.55	0.50	32	0	22	0	46
Discriminate distances	0.57	0.50	32	2	3	1	62
Discriminate colors	0.83	0.38	42	1	2	0	55
Perceive angle of body rotation	0.76	0.43	56	0	11	1	32
Follow the gaze of others	0.40	0.49	25	1	24	1	49
Discriminate two things from three	0.64	0.48	30	2	5	0	63
Discriminate quantities	0.70	0.46	28	2	6	0	64
Think hidden objects are still there	0.58	0.50	18	0	16	0	66
Wash hands/Use litterbox	0.07	0.26	8	0	16	52	24

Table S8. Results of Study 2.

	Estimated age onset					Frequency of explanations			
Ability	*Mean*	*SD*	*Position on the picture scale representing empirical ages*	*T (Compared to the max age of the position)*	*P-value*	*Born able to X*	*X matured with age*	*Learned X on own*	*Taught X by others*
See	0.64	0.97	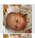 (≤ 0.5)	1.41	0.16	56	39	4	1
Hear	0.65	0.89	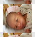 (≤ 0.5)	1.69	0.09	65	20	13	2
Discriminate colors	1.55	1.77	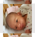 (≤ 0.5)	5.96	< .001	33	33	28	6
Discriminate distances	3.32	2.88	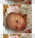 (≤ 0.5)	9.78	< .001	11	25	54	10
Prefer faces to non-faces	1.74	2.14	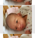 (≤ 0.5)	5.81	< .001	22	26	52	0
Think unsupported objects will fall	2.75	2.44	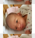 (≤ 0.5)	9.21	< .001	5	10	78	7
Think hidden objects are still there	2.55	2.13	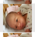 (≤ 0.5)	9.62	< .001	6	16	74	4
Discriminate quantities	3.79	3.42	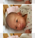 (≤ 0.5)	9.62	< .001	3	24	70	3
Prefer helping to not helping	4.50	4.10	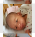 (≤ 0.5)	9.75	< .001	4	11	51	34
Think bigger is mightier	4.19	3.11	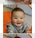 (0.5 - 1)	10.25	< .001	3	13	80	4
Explore unexpected events	5.74	4.01	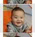 (0.5 - 1)	11.83	< .001	5	28	67	0
Learn from patterns	3.61	2.76	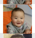 (0.5 - 1)	9.47	< .001	3	17	71	9
Read	4.25	3.74	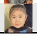 (4 - 7)	-7.34	< .001	3	15	41	41
